# Hypermethylation of Tumor Suppressor Genes Involved in Critical Regulatory Pathways for Developing a Blood-Based Test in Breast Cancer

**DOI:** 10.1371/journal.pone.0016080

**Published:** 2011-01-24

**Authors:** Ramin Radpour, Zeinab Barekati, Corina Kohler, Qing Lv, Nicole Bürki, Claude Diesch, Johannes Bitzer, Hong Zheng, Seraina Schmid, Xiao Yan Zhong

**Affiliations:** 1 Laboratory for Gynecological Oncology, Department of Biomedicine, Women's Hospital, University of Basel, Basel, Switzerland; 2 Department of Breast Surgery, West China Hospital, West China School of Medicine, Sichuan University, Chengdu, China; 3 Department of Obstetrics and Gynecology, Kantonsspital, Liestal, Switzerland; 4 Department of Obstetrics and Gynecology, Women's Hospital, University of Basel, Basel, Switzerland; 5 Laboratory of Molecular Diagnosis of Cancer, Department of Oncology, State Key Laboratory of Biotherapy and Cancer Center, West China Hospital, West China School of Medicine, Sichuan University, Chengdu, China; Deutsches Krebsforschungszentrum, Germany

## Abstract

**Background:**

Aberrant DNA methylation patterns might be used as a biomarker for diagnosis and management of cancer patients.

**Methods and Findings:**

To achieve a gene panel for developing a breast cancer blood-based test we quantitatively assessed the DNA methylation proportion of 248 CpG sites per sample (total of 31,248 sites in all analyzed samples) on 10 candidate genes (*APC*, *BIN1*, *BMP6*, *BRCA1*, *CST6*, *ESR-b*, *GSTP1*, *P16*, *P21* and *TIMP3*). The number of 126 samples consisting of two different cohorts was used (first cohort: plasma samples from breast cancer patients and normal controls; second cohort: triple matched samples including cancerous tissue, matched normal tissue and serum samples). In the first cohort, circulating cell free methylated DNA of the 8 tumor suppressor genes (TSGs) was significantly higher in patients with breast cancer compared to normal controls (*P*<0.01). In the second cohort containing triple matched samples, seven genes showed concordant hypermethylated profile in tumor tissue and serum samples compared to normal tissue (*P*<0.05). Using eight genes as a panel to develop a blood-based test for breast cancer, a sensitivity and specificity of more than 90% could be achieved in distinguishing between tumor and normal samples.

**Conclusions:**

Our study suggests that the selected TSG panel combined with the high-throughput technology might be a useful tool to develop epigenetic based predictive and prognostic biomarker for breast cancer relying on pathologic methylation changes in tumor tissue, as well as in circulation.

## Introduction

Breast cancer is one of the most common types of cancer among women. Localized breast cancer at an early stage has better prognosis and requires less severe treatment with a survival rate of 98% [1]. However, diagnosis after tumor metastasis lowers the survival rate to 27% [2]. This highlights the importance of early breast cancer detection which is dependent on sensitive and specific screening methods. The traditional triple test for breast cancer diagnosis includes physical examination, mammography and aspiration cytology. Unfortunately, all these methods are not sensitive enough in identifying breast cancer in early stages [1], [3]. A minimally invasive screening test beside the triple test, or prior to biopsy, would lead to greater sensitivity.

It is well recognized that solid malignant tumors release significant amounts of DNA into the systemic circulation through cellular necrosis or apoptosis [4]. The presence of cell-free DNA (cfDNA) in plasma and serum has been known for over 60 years. Quantitative alteration of circulating cfDNA has been observed in several cancers, such as prostate cancer [5], lung cancer [6], pancreatic cancer [7], and breast cancer [8]. The tumor released DNA in circulation might serve as biomarker for cancer [8].

Aberrant promoter methylation pattern of tumor suppressor genes (TSGs) is known to be a frequent and early event in carcinogenesis [9], [10], [11], [12]. Tumor-specific methylated DNA alterations have been found in the circulation of patients with different types of cancer [12], [13]. The analysis of the methylation patterns of cfDNA by a blood-based test might enable to distinguish between benign and malignant tumors for diagnosis and surveillance of patients [12].

The SEQUENOM's EpiTYPER™ assay is a high-throughput methylation quantification method which relies on matrix-assisted laser desorption/ionization time-of-flight mass spectrometry (MALDI-TOF MS) [14]. The sensitivity, specificity and assay concept of the method have been previously described by ulterior studies [14], [15], [16], [17], [18], [19], [20]. Recently, we analyzed the methylation profiles of more than 42,528 CpG sites on 22 genes of which 10 were shown to be hypermethylated genes (*APC*, *BIN1*, *BMP6*, *BRCA1*, *CST6*, *ESR-b*, *GSTP1*, *P16*, *P21* and *TIMP3*) in cancerous breast tissue in comparison with matched normal tissue [17]. These 10 hypermethylated genes were considered as methylation signature of breast cancer and were used in this study for further investigations to develop an epigenetic blood-based assay for breast cancer.

In the present study, to achieve a reliable gene panel for developing a blood-based test, we quantitatively assessed the DNA methylation profile of 10 breast cancer candidate genes using MALDI-TOF MS in two different cohorts of patients with breast cancer on large-scale CpG sites.

## Materials and Methods

The study was performed at the Laboratory for Gynecological Oncology, Department of Biomedicine, Women's Hospital, Basel and approved by the local institutional review board (Ethic commission beider Basel). Written consent forms were collected from all patients who were involved in this study.

### Sampling and pathological classification

In total 126 samples were used in this study. For analysis we divided these samples in two different cohorts. The first cohort consisted of 36 plasma samples of breast cancer patients and 30 plasma samples of healthy non-relative women. The second cohort consisted of 60 triple samples (cancerous tissue, matched normal tissue and serum samples) from 20 patients with non-familial breast cancer. Staging and grading was evaluated according to the WHO histological classification. Breast cancer characteristics, such as staging, histological grading, and hormone receptor expression from the two different cohorts are summarized in Table 1.

**Table 1:** Clinical characteristics of patients in the two study cohorts.

Sample type	Total no. of patients	Age mean ± S.D. (range)	Pathologic stage		No. of patients with lymph node involvement	No. of patients with metastasis	Histological grade			ER		PR	
			Early*	Late**			1	2	3	Positive	Negative	Positive	Negative
**Plasma samples**	36	67±13.4 (38–89)	27	9	19	0	11	18	7	28	8	23	13
**Triple samples**	20	50±11.7 (33–77)	12	8	13	0	0	5	15	16	4	10	10

*The pathologic stage<III was considered as “Early stage”.

**The pathologic stage III and IV was considered as “Late stage”.

ER: Estrogen receptor; PR: Progesterone receptor.

### Isolation of circulating cfDNA from plasma and serum

A total of 20 mL blood samples were collected in both EDTA tubes (for plasma) and EDTA-free tubes (for serum) and processed immediately after collection. The plasma samples were centrifuged at 1,600×g (10 min), and supernatant was carefully transferred into 2 mL microtubes. Samples were centrifuged in a microcentrifuge at full speed (10 min), and supernatants were stored at −80°C until analysis was performed. The serum tubes were coagulated during approximately 1h, after which the serum was harvested and stored using the above mentioned procedure.

DNA extraction was performed from 25–50 mg of frozen tissue and 600 µL of plasma and serum using the High Pure PCR Template Preparation Kit (Roche Diagnostics, Mannheim, Germany) and eluted in a final volume of 100 µL. The median quantity of extracted cfDNA in plasma and serum were 5.7 ng/µL (range 2.6 to 12.1) and 7.1 ng/µL (range 5.4 to 14.8) respectively. The median quantity of extracted DNA from frozen tissue was 65.7 ng/µL (range 28.3 to 186.1).

Before performing the methylation analysis, we quantified the yield of extracted DNA by quantitative PCR for the *GAPDH* (glyceraldehyde 3-phosphate dehydrogenase) gene. The good quality of the extracted DNA allowed successful amplification and quantification of the *GAPDH* gene in all samples (data not shown).

### Bisulfite Treatment

To perform bisulfite conversion of the target sequence, the Epitect® Bisulfite Kit (QIAGEN AG, Basel, Switzerland) was used according to the manufacturer's protocol.

### Primer design and PCR-tagging for EpiTYPER™ assay

We used previously designed and tagged primers (reverse primer with T7-promoter tag and forward primer with 10mer tag sequence as balance) for the 10 candidate genes [17]. Selected amplicons were mostly located in the promoter regions, or started from the promoter and partially covered the first exon [17]. For the PCR on bisulfite-treated genomic DNA (gDNA), the following PCR conditions were used: 1×: 95°C for 10 min; 48×: 95°C for 30s, Ta for 40s, 72°C for 1 min; 1× 72°C for 5 min. The PCR cocktail was: 2µL DNA (2.00µL of at least 10 ng/µL DNA for a final concentration of 2ng/µL per reaction) in a 10µL total volume using 1pmol of each primer, 200µM dNTP, 0.2 unit Hot Start Taq DNA polymerase, 1.5mM MgCl2 and the buffer supplied with the enzyme.

### *In vitro* transcription and T-cleavage assay

*In vitro* transcription and T-cleavage assay were assessed according to the previously published methods [14], [16], [17]. Briefly, unincorporated dNTPs were removed by shrimp alkaline phosphatase (SAP; SEQUENOM, Inc., San Diego, CA) treatment. Typically, 2 µL of the PCR product were used as template for the transcription reaction. Twenty units of T7 R&DNA polymerase (Epicentre, Madison, WI) were used to incorporate dTTP in the transcripts. Ribonucleotides and dNTPs were used at concentrations of 1 mmol/L and 2.5 mmol/L, respectively. In the same step, RNase-A (SEQUENOM Inc., San Diego, CA) was added to cleave the *in vitro* transcripts (T-cleavage assay). Samples were diluted with H_2_O to a final volume of 27 µL. Conditioning of the phosphate backbone was achieved by adding 6 mg of Clean Resin (SEQUENOM) before performing MALDI-TOF MS analysis.

### Mass spectrometry

Twenty-two nanoliters of the RNase-A treated product were robotically dispensed onto silicon matrix preloaded chips (SpectroCHIP; SEQUENOM, San Diego), the mass spectra were collected using a MassARRAY® Compact MALDI-TOF (SEQUENOM) and spectra's methylation proportion were generated by the EpiTYPER™ software v1.0 (SEQUENOM, San Diego).

### Statistical methods

Data analysis was performed using the PASW Statistics software v.18. The Shapiro-Wilk and Kolmogorov-Smirnov tests were used for data distribution analysis. Both tests similarly demonstrated that our data set was not normally distributed (Shapiro-Wilk test; *P*<0.001 and Kolmogorov-Smirnov test; *P*<0.001). Quantitative methylation proportion of 10 genes was analyzed in two different study cohorts. Using two-way hierarchical cluster analysis, the most variable CpG sites for each gene were clustered based on pair-wise Euclidean distances and linkage algorithm for all studied samples according to the previously developed method by Gene Expression Statistical System (GESS) version 7.1.19 (NCSS, Kaysville, Utah, USA) [16], [17], [18]. The Mann-Whitney U test was used to compare the promoter methylation between study groups and also with clinicopathological parameters. The non-parametric Spearman's rho test was used to find out the correlation of methylation proportion in serum versus tumor and normal samples. Three dimensional principal component analysis (PCA) was accessed for both different cohorts based on the methylation proportion of 10 studied genes to transform a number of possibly correlated variables into a smaller number of uncorrelated variables.

## Results

### Quantitative methylation profiling of the 10 studied genes

**Figure 1:**
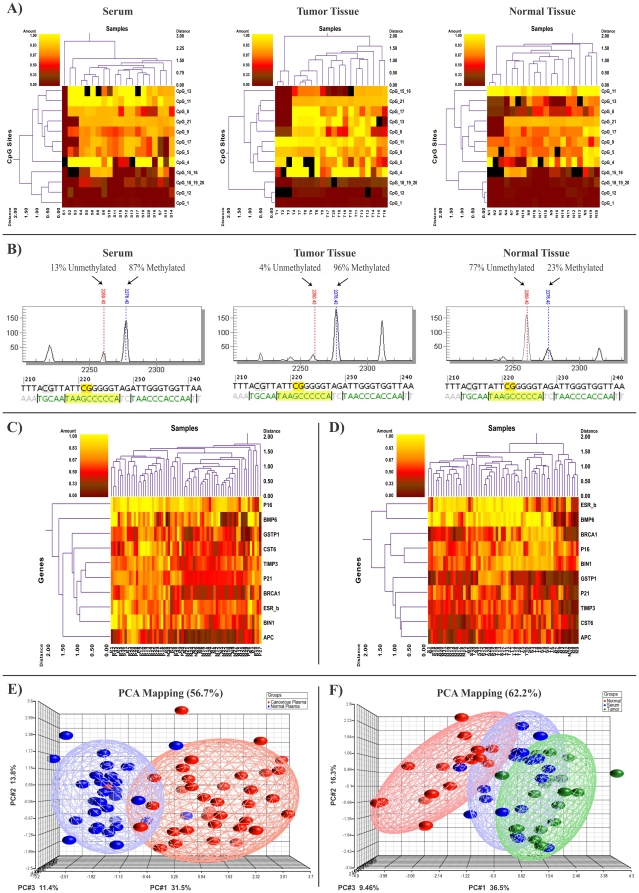
Methylation profiling of 10 candidate genes in two studied cohorts. A) An example of high-throughput methylation analysis of CpG sites for the *BRCA1* gene for the 60 triple samples (cancerous tissue, matched normal tissue and serum samples). The complete data for the other genes is summarized in Dataset S1. B) Peaks show percentage of methylation extent obtained from an informative CpG site of *BRCA1* gene with a significant difference between serum and tumor with normal tissue in a triple case. C) Double dendrogram profiles the mean methylation proportion of all 10 studied genes in plasma samples from breast cancer patients and normal subjects. D) Double dendrogram profiles the mean methylation proportion of all 10 studied genes in triple matched samples. E) PCA mapping of the mean methylation proportion of analyzed genes in plasma samples. F) PCA mapping of the mean methylation proportion of analyzed genes in triple matched samples.

In this study, we analyzed the methylation proportion of 10 breast cancer candidate genes in 126 different samples consisting of two different cohorts (36 plasma samples from patients with breast cancer and 30 plasma samples from normal controls, as well as 60 triple matched samples containing cancerous tissue, normal tissue and serum from 20 breast cancer patients). For all of the studied genes one amplicon per gene was analyzed and all amplicons contained CpG rich islands (with the number of CpG sites higher than 20) (Table 2). In total, we assessed 10 amplicons, containing 248 CpG sites per sample (total of 31,248 sites in all analyzed samples) (Table 2; Fig. 1; Dataset S1). From several analyzed CpG sites per amplicon few of them could represent valuable differences in the studied cases which were considered as informative CpG sites (Table 2). The mean methylation quantity of the informative CpG sites per each gene was used to figure out the methylation proportion of the candidate genes (Dataset S1).

**Table 2:** High-throughput methylation analysis of CpG sites per amplicon for the 10 studied genes.

Genes	Amplicon size (bp)	Total No. of CpG sites in amplicon	No. of analyzed CpG sites in amplicon	No. of informative CpG sites in amplicon	No. of analyzed CpG sites per amplicons	
					Single sites	Composite sites
*APC*	420	26	18	12	12	6
*BIN1*	330	32	18	12	3	15
*BMP6*	397	37	30	9	11	19
*BRCA1*	413	30	15	7	10	5
*CST6*	445	49	27	6	15	12
*ESR-b (ER beta)*	374	30	24	6	7	17
*GSTP1*	381	23	17	8	10	7
*P16 (CDKN2A)*	580	62	36	14	13	23
*P21 (CDKN1A)*	419	30	19	8	10	9
*TIMP3*	441	51	44	14	11	33

The *in silico* digestion was performed for the T-cleavage assay. The percentage of total CpG sites in the amplicon is divided into single sites (single CpG sites) and composite sites (two or more adjacent CpG sites fall within one fragment, or when fragment masses are overlapping).

#### Methylation proportion of candidate genes in plasma samples

**Figure 2:**
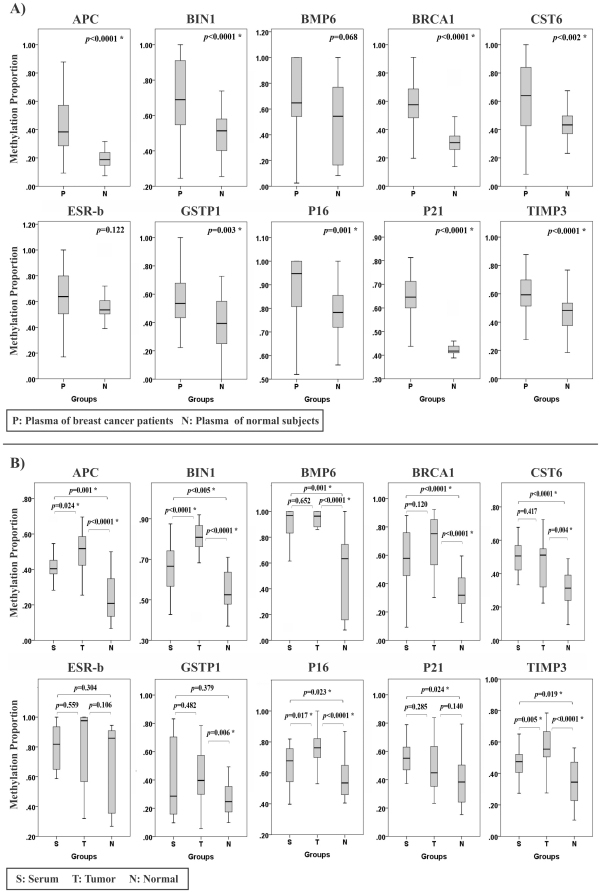
Comparison between quantitative methylation analyses of 10 candidate genes. A) Thirty six plasma samples of breast cancer patients and 30 plasma samples of normal subjects as control. B) Triple matched samples from 20 breast cancer patients. (* significant difference; Mann-Whitney U Test).

Methylation proportion of each CpG site is given on a scaling 0 to 100 percent. Using two-way hierarchical cluster analysis, we found different methylation pattern of the candidate genes in plasma samples between patients with breast cancer and normal controls (Fig. 1; Dataset S1). Cell free methylated DNA levels of 8 genes (*APC*, *BIN1*, *BRCA1*, *CST6*, *GSTP1*, *P16*, *P21* and *TIMP3*) were significantly higher in the plasma samples from patients with breast cancer in comparison with those from normal controls (*P*<0.01), while the other two genes *BMP6* and *ESR-b* showed the same tendency but was not significant (*P*>0.05) (Fig. 2a; Dataset S1). PCA mapping based on the mean methylation proportion of eight genes in the cohort of plasma samples showed a number of possibly correlated samples into a smaller number of uncorrelated samples (Fig. 1E).

#### Methylation proportion of candidate genes in triple matched samples

To confirm that hypermethylation of cell free DNA in circulation of breast cancer patients are derived from tumor tissue, triple matched samples including cancerous tissue, matched normal tissue and serum samples were analyzed. Hierarchical clustering showed significant hypermethylation patterns for serum and tumor tissue compared with normal tissue for seven genes (*APC*, *BIN1*, *BMP6*, *BRCA1*, *CST6*, *P16* and *TIMP3*). The *GSTP1* gene was significantly hypermethylated in tumor tissue and *P21* gene was mostly hypermethylated in serum samples compared to the normal tissue. For the *ESR-b* gene no significant differences in the methylation extent was observed between studied groups (Fig. 2b). PCA mapping based on the mean methylation proportion of seven genes in the cohort of triple matched samples showed a number of possibly correlated samples into a smaller number of uncorrelated samples (Fig. 1F).The complete methylation quantification data for 10 studied genes is summarized in dataset S1.

A correlation study of the methylation proportion of DNA in serum versus cancerous and normal breast tissue revealed a correlation between tumor tissue and serum for *BMP6*, *BRCA1*, *CST6*, *GSTP1*, *P16* and *TIMP3* genes but not with the matched normal tissue (Dataset S2).

### Sensitivity and specificity of a blood based assay to distinguish tumor derived hypermethylated DNA with non-hypermethylated DNA

**Figure 3:**
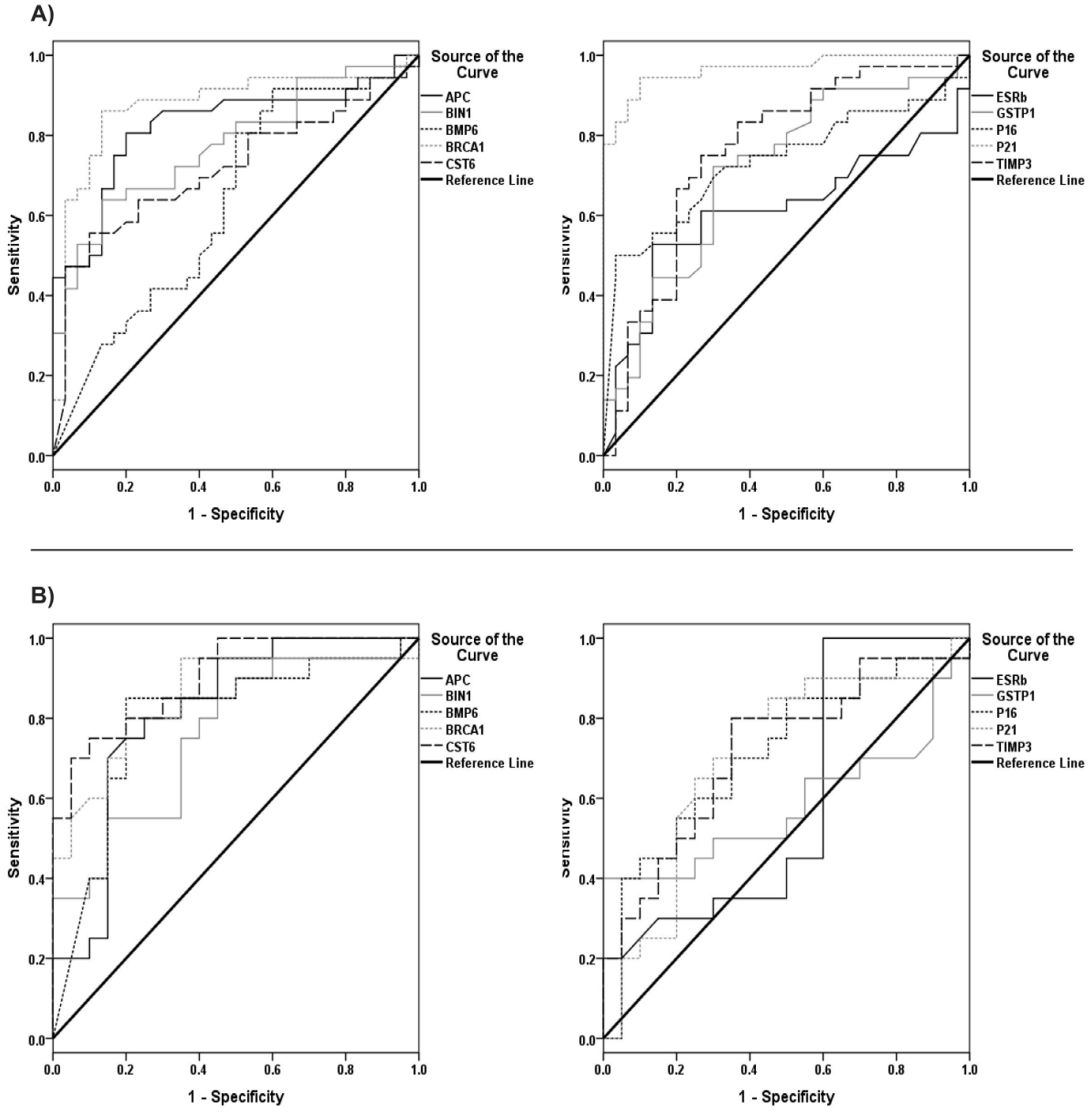
ROC curve analysis using cfDNA for discriminating between cancerous and normal samples based on methylation patterns of 10 candidate genes. A) ROC curves of cfDNA to discriminate between plasma sample of breast cancer patients and plasma samples of normal subjects. B) ROC curves of cfDNA to discriminate between serum with and matched normal tissue samples.

To find a reliable gene panel which could serve as sensitive and specific blood-based methylation test, gene coverage analysis was assessed for 8 genes with significant different methylation pattern between cancerous and normal plasma. To evaluate the applicability of circulating cfDNA as a biomarker for breast cancer, receiver operating characteristic (ROC) curve analysis was used. Cut-off points, sensitivity, area under the curve (AUC) and confidence interval were calculated for each gene respectively based on at least 90% specificity (Figure 3; Table 3). The methylation quantity over cut-off points were considered as hypermethylation per gene to calculate the methylation frequency in all studied cases (Table 4). As blood-based marker, our designed panel could cover 91.7% of the plasma samples (92.6% for early stage and 88.9% for late stage of breast cancer) and also covered 95% of serum samples (91.7% for early stage and 100% for late stage of breast cancer) (Table 4).

**Table 3:** ROC curve analysis of plasma and serum samples based on methylation proportion of the 10 genes.

Sample type	Genes	Specificity (%)	Sensitivity (%)	AUC*	Asymptotic 95% confidence interval (Lower - Upper Bound)	Cut-off points** (methylation quantification)
**Plasma**	*APC*	90	50	0.824	0.720–0.928	0.42
	*BIN1*	90	53	0.776	0.665–0.888	0.67
	*BMP6*	90	30	0.631	0.493–0.768	0.71
	*BRCA1*	90	75	0.874	0.780–0.967	0.57
	*CST6*	90	56	0.718	0.592–0.843	0.58
	*ESR-b (ER beta)*	90	31	0.611	0.471–0.752	0.76
	*GSTP1*	90	35	0.712	0.585–0.838	0.66
	*P16 (CDKN2A)*	90	50	0.732	0.608–0.856	0.91
	*P21 (CDKN1A)*	90	88	0.965	0.915–1.000	0.65
	*TIMP3*	90	35	0.761	0.640–0.882	0.68
**Serum**	*APC*	90	25	0.814	0.676–0.951	0.46
	*BIN1*	90	40	0.757	0.608–0.907	0.69
	*BMP6*	90	45	0.795	0.647–0.943	0.93
	*BRCA1*	90	60	0.854	0.730–0.977	0.70
	*CST6*	90	75	0.870	0.809–0.991	0.53
	*ESR-b (ER beta)*	90	30	0.595	0.409–0.781	0.92
	*GSTP1*	90	40	0.581	0.393–0.769	0.43
	*P16 (CDKN2A)*	90	40	0.710	0.546–0.874	0.69
	*P21 (CDKN1A)*	90	25	0.709	0.540–0.877	0.66
	*TIMP3*	90	35	0.717	0.557–0.878	0.48

*AUC: area under the curve.

**Cut-off points were calculated according to 90% specificity.

**Table 4:** Frequency and coverage of promoter methylation in plasma and serum cfDNA.

Type of tumor	Promoter methylation frequency in *plasma*			Promoter methylation frequency in *serum*		
	Genes	Methylation frequency	Coverage^a^	Genes	Methylation frequency	Coverage^a^
Samples with early stage* of cancer	*APC*	48.1%	92.6%	*APC*	25%	91.7%
	*BIN1*	55.6%		*BIN1*	41.7%	
	*BRCA1*	48.1%		*BRCA1*	58.3%	
	*CST6*	55.6%		*CST6*	41.7%	
	*GSTP1*	29.6%		*GSTP1*	8.4%	
	*P16 (CDKN2A)*	59.2%		*P16 (CDKN2A)*	41.7%	
	*P21 (CDKN1A)*	40.7%		*P21 (CDKN1A)*	16.7%	
	*TIMP3*	33.3%		*TIMP3*	25%	
Samples with late stage** of cancer	*APC*	44.4%	88.9%	*APC*	25%	100%
	*BIN1*	44.4%		*BIN1*	12.5%	
	*BRCA1*	55.5%		*BRCA1*	12.5%	
	*CST6*	44.4%		*CST6*	12.5%	
	*GSTP1*	22.2%		*GSTP1*	75%	
	*P16 (CDKN2A)*	22.2%		*P16 (CDKN2A)*	37.5%	
	*P21 (CDKN1A)*	33.3%		*P21 (CDKN1A)*	37.5%	
	*TIMP3*	22.2%		*TIMP3*	62.5%	
All analyzed samples	*APC*	47.2%	91.7%	*APC*	25%	95%
	*BIN1*	52.8%		*BIN1*	30%	
	*BRCA1*	50%		*BRCA1*	40%	
	*CST6*	52.8%		*CST6*	30%	
	*GSTP1*	27.8%		*GSTP1*	35%	
	*P16 (CDKN2A)*	50%		*P16 (CDKN2A)*	40%	
	*P21 (CDKN1A)*	38.9%		*P21 (CDKN1A)*	25%	
	*TIMP3*	30.6%		*TIMP3*	40%	

a Coverage: percentage of cases with methylation in at least one gene in the given panel (i.e., coverage of 100% means that all samples had methylation of at least one gene in the panel).

*The pathologic stage<III was considered as “Early stage”.

**The pathologic stage III and IV was considered as “Late stage”.

### Relationship between promoter methylation and clinicopathological parameters

In this study, associations between the promoter methylation of the 10 studied genes in cfDNA of breast cancer patients and clinicopathological parameters, such as age, histological grade, pathologic stage, lymph node involvement and receptor status were analyzed (Dataset S3).

In plasma samples, promoter hypermethylation of *GSTP1* gene was significantly correlated with higher age (≥50), hypermethylation of *P16* gene was correlated with pathologic early stage of cancer and hypermethylation of *BMP6* gene was correlated with lymph node involvement (*P*<0.05) (Dataset S3). In normal cases, there was no significant correlation between methylation proportion of candidate genes and clinicopathological parameters.

In triple matched samples, promoter hypermethylation extent of four TSGs in serum samples showed correlation with clinical parameters (*APC* with histological grade G2; *BMP6* and *CST6* with lymph node involvement; *TIMP3* with pathological late stage and lymph node involvement) (*P*<0.05) (Dataset S3).

### Comparison of methylation proportion with recognition sites of well-known transcription factor regions

**Figure 4:**
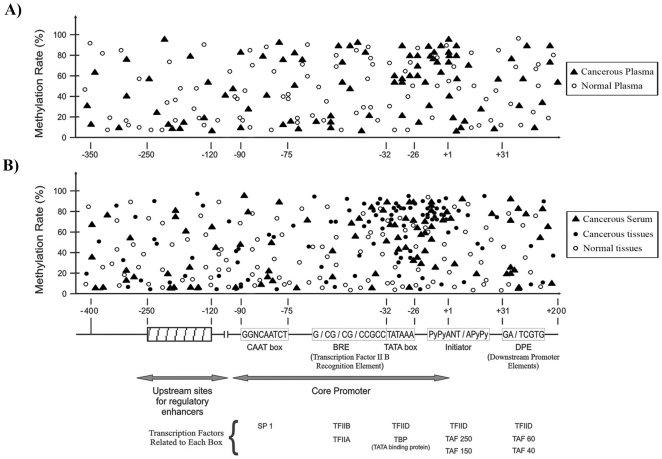
Comparison of the mean methylation proportion and approximate position of informative CpG sites in the range of −400 to +200 according to the recognition sites of the transcription factors in the 10 candidate genes. A) Comparison of methylation proportion in 36 plasma samples of breast cancer patients and 30 plasma samples of normal subjects. B) Comparison of methylation proportion in 60 triple samples (cancerous breast tissue, matched normal tissue and serum samples) from 20 breast cancer patients. (Dots in the map are corresponding to the mean methylation quantity of each CpG site in all analyzed cases).

The methylation proportion and localization of each CpG site in the range of −400 to +200 was schematically compared to the consensus sequences of well-known transcription factors (upstream sites for regulatory enhancers, CAAT box, GC box, transcription factor II-B recognition elements, TATA box, initiation site of transcription and downstream promoter elements) for the both cohorts (Fig. 4).

Hypermethylated CpG sites in the plasma sample from breast cancer patients were almost located between TATA box and initiator, however, in plasma of normal subjects hypermethylated CpGs were randomly distributed and did not show significant association between location of the CpG sites and conserved sequences (Fig. 4a).

The analysis of triple matched samples revealed that the hypermethylated CpG sites in tumor tissue and serum samples were mostly located in a range of −40 to +1 (TATA box and initiator) and −26 to +1 (TATA box), respectively. While in normal samples the CpG sites were differentially methylated and located randomly in the 5′UTRs of the studied genes (Fig. 4b).

## Discussion

In this study, we assessed the methylation proportion of more than 31,248 CpG sites on 10 breast cancer candidate genes in 126 different samples consisting of two different cohorts. Using hierarchical clustering in the plasma samples cohort, we found significant promoter hypermethylation of eight genes (*APC*, *BIN1*, *BRCA1*, *CST6*, *GSTP1*, *P16*, *P21* and *TIMP3*) in patients' plasma of breast cancer patients compared with plasma of normal subjects (Fig. 2a). To proof that hypermethylation pattern in plasma cfDNA of breast cancer patients is derived from tumor tissue, triple matched samples including breast cancerous tissue, matched normal tissue and serum samples were analyzed. Two-way hierarchical clustering in serum and tumor tissue showed significant hypermethylation patterns of seven genes (*APC*, *BIN1*, *BMP6*, *BRCA1*, *CST6*, *P16* and *TIMP3*) compared with normal tissue (Fig. 2b; Dataset S1). This data revealed the potential of the candidate genes for developing a blood-based test as a predictive and prognostic biomarker for breast cancer.

The pathway analysis showed the involvement of the 10 candidate genes in cell cycle and DNA repair (*BRCA1*, *P16* and *P21*), invasion and metastasis (*CST6* and *TIMP3*), cell proliferation (*ESR-b*), signal transduction (*APC*, *BIN1* and *BMP6*) and cell detoxification (*GSTP1*), and highlighted their role in breast carcinogenesis. Approximately one-third of the differentially methylated 5′UTRs are inversely correlated with transcription in normal tissue [17], [21]. Similarly, present study showed accumulation of hypermethylated CpG sites of 10 studied genes nearby TATA box and initiator (−40 to +1) in the cancerous and serum samples, however, in normal samples the CpG sites were differentially methylated and located randomly in the 5′-UTRs (Fig. 4). This data verified that hypermethylation of critical part of promoter regions might be the major mechanism for transcription alterations leading to silencing or down regulation of candidate genes. Also hypermethylation of several genes in the same pathway might contribute to tumor aggressiveness.

It has been estimated that more than 90% of the total circulating cfDNA is derived from tumor tissue [22], [23]. Several studies reported tumor related genetic and epigenetic alterations in serum and plasma cfDNA of breast cancer patients [24], [25], [26], [27], but studies comparing methylation patterns in tumor and serum DNA in early or late stages of breast tumorigenesis are limited. According to the origin of plasma or serum cfDNA which is released during cell necrosis or apoptosis, it appears that serum tends to contain more DNA than plasma. However, some of this DNA in serum could be due to DNA contamination derived from leukocytes [28]. In our study, there was significant concordance regarding the methylation patterns of seven analyzed genes in serum sample with tumor tissue (Dataset S1 & 2). This result suggested that cancer specific methylation changes in plasma and serum could be used in developing blood-based tests, which could be applied for risk assessment, earlier diagnosis and monitoring of cancers.

Methylation changes in the process of tumorigenesis are often very heterogeneous and still no single gene has been found to be methylated in all breast cancer types. Therefore it is necessary to use a panel of genes as biomarkers to screen certain type of cancer. Different studies have shown a wide range of gene panels according to the frequently methylated genes in different type of cancer including breast cancer. The coverage and sensitivity of reported panels to detect different types of breast cancer, ranges from 40 to 90% depended on the selected genes [10], [17], [26], [29], [30], [31], [32], [33]. In the recent study, applying the eight genes panel could achieve to 91.7% of coverage in sensitive methylation quantification for plasma and 95% for serum samples with more than 90% specificity in both studied cohorts (Table 4).

The variability of the reported gene panels in different studies makes it difficult to compare or combine them and to interpret how promoter methylation would serve as biomarker [34]. The inclusion of genes that may have a key role in breast cancer might help to improve the specificity of a gene panel. Our finding highlights the necessity of using different genes in one panel which increases the coverage of detected cases nearly to 100% (Table 4).

The correlation analysis between methylation proportion of 10 candidate genes in plasma of breast cancer patients and clinicopathological parameters revealed significant correlation of *GSTP1* hypermethylation with higher age (≥50), *P16* hypermethylation with early stage of breast cancer and *BMP6* with lymph node involvement. In the serum samples, promoter hypermethylation of some studied genes was correlated with clinical parameters (*APC* with histological grade G2; *TIMP3* with late stage of breast cancer; *BMP6*, *CST6*, and *TIMP3* with lymph node involvement) (Dataset S3). This data might require further validation by using bigger sample size cohorts.

Technically, we quantified methylation proportion of the candidate genes in cfDNA derived from plasma and serum using T-cleavage assay on MALDI-TOF MS. According to the origin of cfDNA in plasma or serum which is released during cell necrosis or apoptosis, the majority of isolated cfDNA should be poor with regard to quantity and quality and it is difficult to deal with long amplicons for further downstream experiments [5], [18], [35]. We could overcome this limitation of dealing with fractionated, low concentrated and poor quality DNA, using a specialized re-amplification strategy for performing high-throughput methylation analysis on MALDI-TOF MS based on our established method [18].

Presented data is promising to design a gene panel and develop a blood-based screening method for breast cancer which relies on pathologic methylation changes. Tissue specific and blood-based methylation markers might provide valuable information as prognostic and predictive markers for breast cancer, as well as for developing novel targeted therapeutic strategies.

### Additional information

The complete data for high-throughput methylation analysis of informative CpG sites in 10 breast cancer-related genes, including: gene location, amplicon size and two-way hierarchical cluster analysis of two different studied cohorts are illustrated in dataset S1. Scatterplot Matrix (SPLOM) analysis and correlation of the methylation extent of DNA from different are summarized in dataset S2. Correlation study between promoter methylation extent and clinicopathological parameters is shown in dataset S3.

## Supporting Information

Dataset S1 Double dendrogram of analyzed genes: Two-way hierarchical cluster analysis of 36 plasma samples from breast cancer patients and 30 plasma samples of normal subjects.Comparison of informative CpG sites in two groups of plasma samples.Double dendrogram of analyzed genes: Two-way hierarchical cluster analysis of 60 triple samples (breast cancerous tissue, matched normal tissue serum and samples) from 20 breast cancer patients
(PDF)

Dataset S2 Scatterplot Matrix (SPLOM) analysis for mean methylation proportion of 10 genes in triple samples from 20 breast cancer patients (breast cancerous tissue, matched normal tissue and serum samples).Correlation study of the mean methylation proportion of informative CpG sites for ccfDNA in serum versus tumor and normal samples (S: Serum, N: Normal, T: Tumor).Scatterplot Matrix (SPLOM) analysis for mean methylation proportion of 10 genes in 36 plasma samples of breast cancer patients and 30 plasma samples of normal subjects
(PDF)

Dataset S3 Correlation study between promoter methylation of 10 studied genes and clinicopathological parameters in 36 plasma and 20 serum samples
(PDF)
