# Supplementary material for: Hypermethylation of Tumor Suppressor Genes Involved in Critical Regulatory Pathways for Developing a Blood-Based Test in Breast Cancer
Source: PLoS One. 2011 Jan 24;6(1):e16080. doi: 10.1371/journal.pone.0016080 (PMC3025923; doi:10.1371/journal.pone.0016080)
Supplement: Dataset S1 — Double dendrogram of analyzed genes: Two-way hierarchical cluster analysis of 36 plasma samples from breast cancer patients and 30 plasma samples of normal subjects.Comparison of informative CpG sites in two groups of plasma samples.Double dendrogram of analyzed genes: Two-way hierarchical cluster analysis of 60 triple samples (breast cancerous tissue, matched normal tissue serum and samples) from 20 breast cancer patients (PDF) [file pone.0016080.s001.pdf]

Dataset S1

High-throughput methylation analysis of CpG sites in 10 candidate genes related to breast cancer.

APC Gene

Gene ID	Alternate gene name	locus	Function	Methylation effect on breast cancer
324	adenomatous polyposis coli	5q21-q22	Cell adhesion, signal transduction, stabilization of the cytoskeleton, regulation of cell cycle and apoptosis	direct

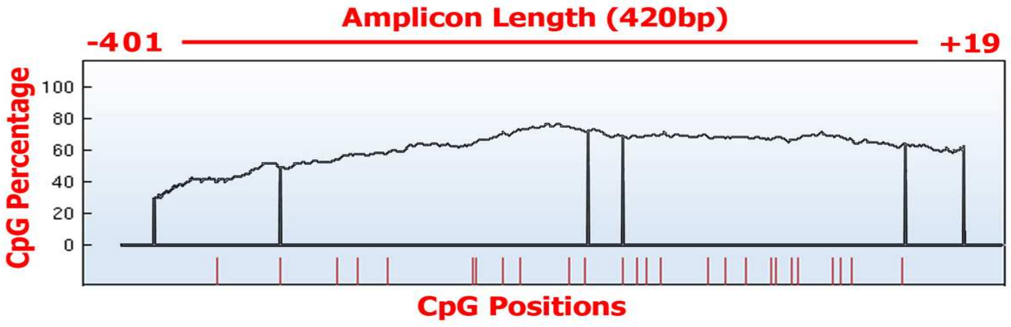

Breast Cancerous Plasma   Normal Plasma

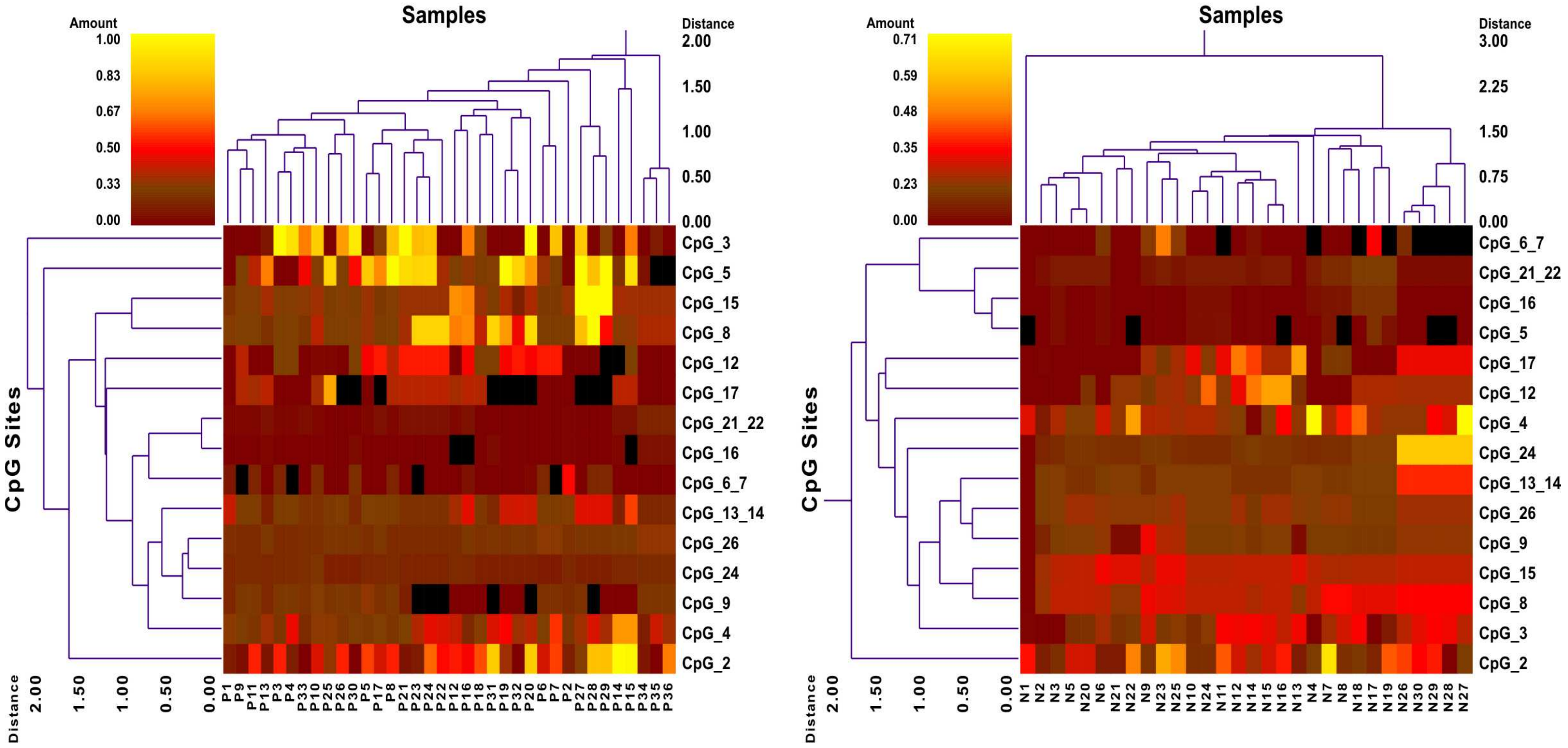

Double dendrogram of *APC* gene: Two-way hierarchical cluster analysis of 36 plasma samples from breast cancer patients and 30 plasma samples of normal subjects. (Red clusters indicate 0% methylated, yellow clusters indicate 100% methylated, color gradient between red and yellow indicates methylation ranging from 0-100, and black clusters indicate not analyzed CpG sites).

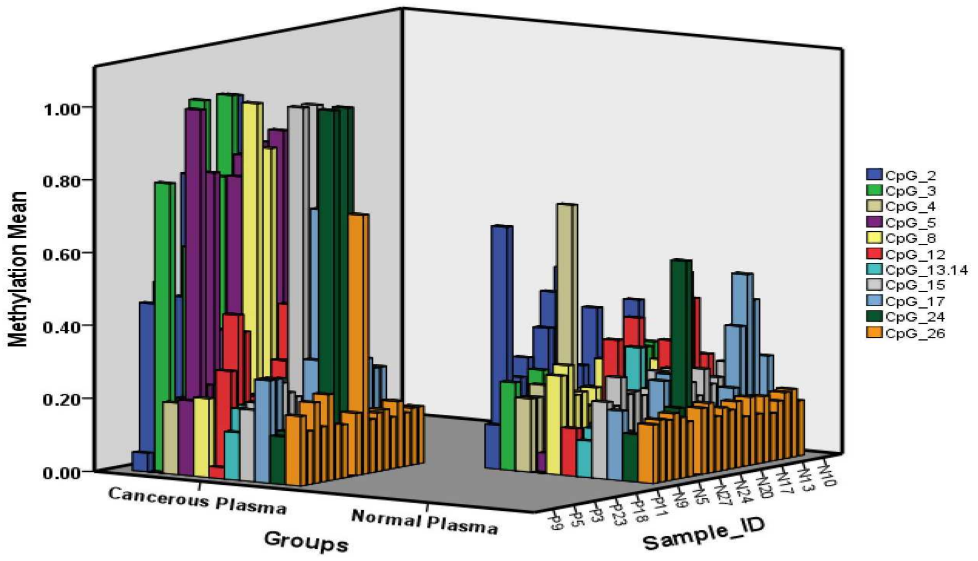

Comparison of informative CpG sites in two groups of plasma samples.

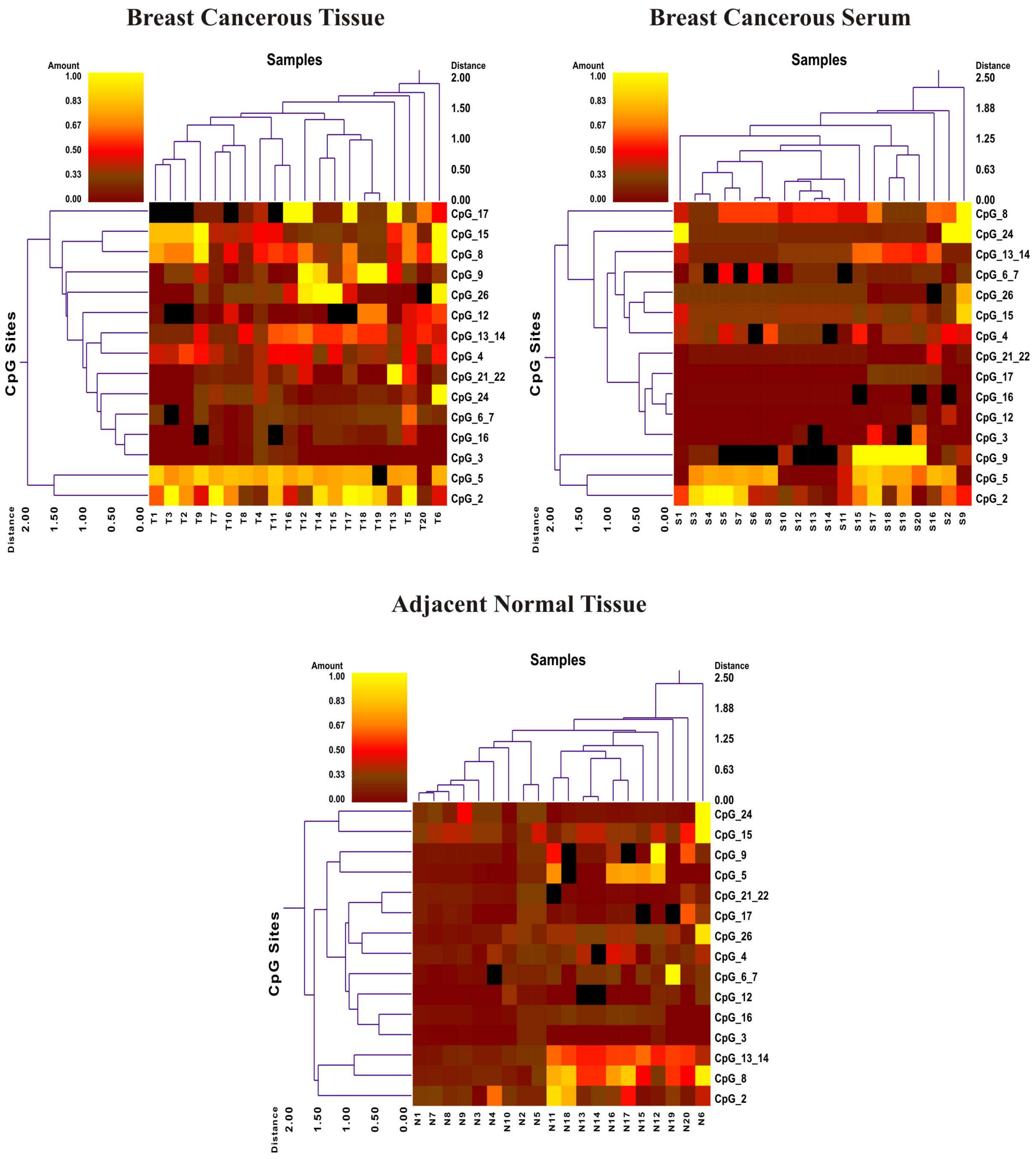

Double dendrogram of *APC* gene: Two-way hierarchical cluster analysis of 60 triple samples (breast cancerous tissue, matched normal tissue serum and samples) from 20 breast cancer patients. (Red clusters indicate 0% methylated, yellow clusters indicate 100% methylated, color gradient between red and yellow indicates methylation ranging from 0-100, and black clusters indicate not analyzed CpG sites).

BIN1 Gene

Gene ID	Alternate gene name	locus	Function	Methylation effect on breast cancer
274	Bridging integrator 1	2q14	Encodes several isoforms of a nucleocytoplasmic adaptor protein, one of which was initially identified as a MYC-interacting protein with features of a tumour suppressor.	direct

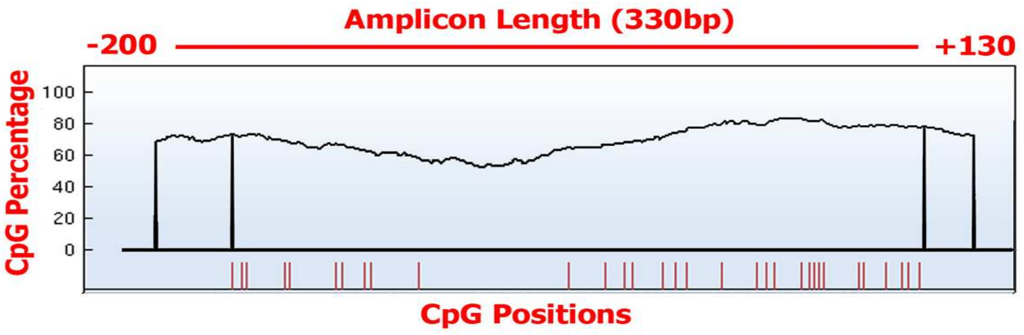

Breast Cancerous Plasma | Normal Plasma

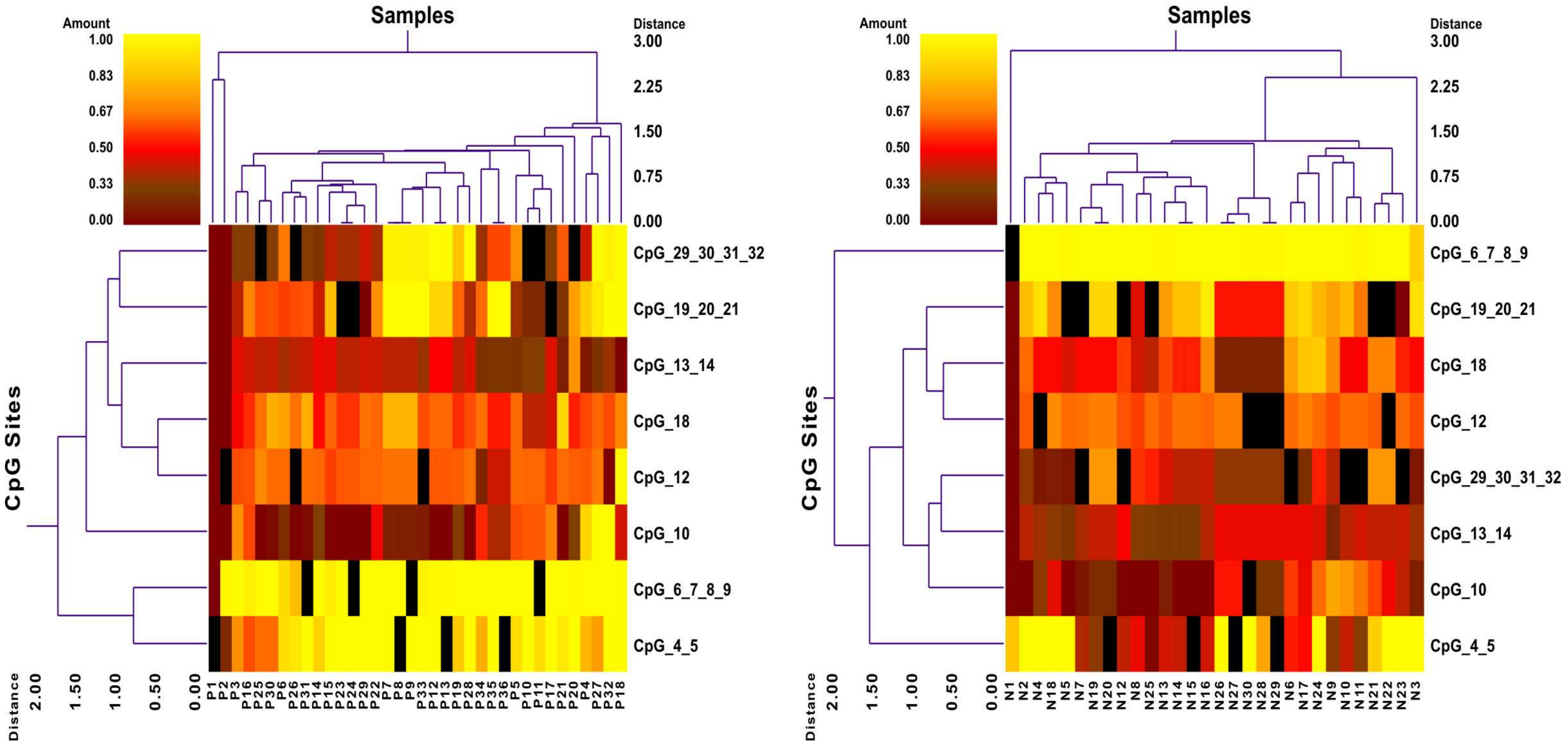

Double dendrogram of *BIN1* gene: Two-way hierarchical cluster analysis of 36 plasma samples from breast cancer patients and 30 plasma samples of normal subjects. (Red clusters indicate 0% methylated, yellow clusters indicate 100% methylated, color gradient between red and yellow indicates methylation ranging from 0-100, and black clusters indicate not analyzed CpG sites).

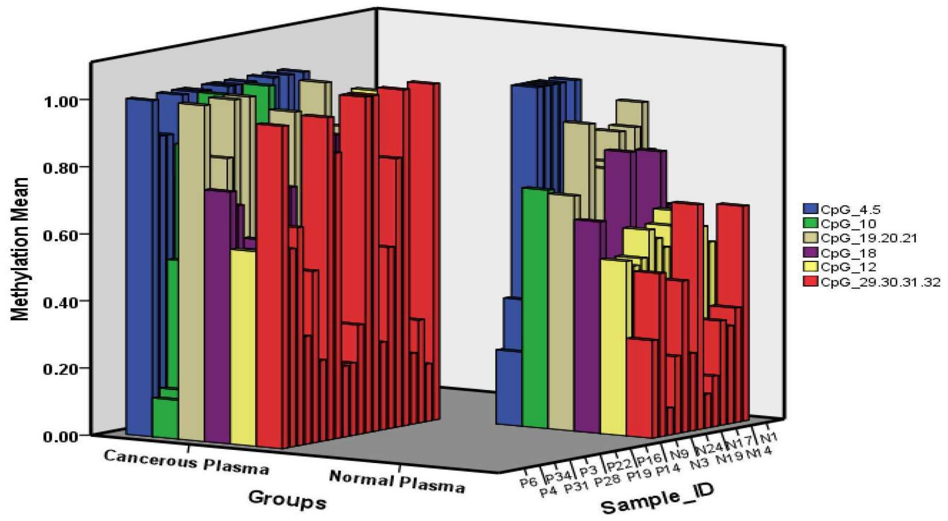

Comparison of informative CpG sites in two groups of plasma samples.

Breast Cancerous Tissue

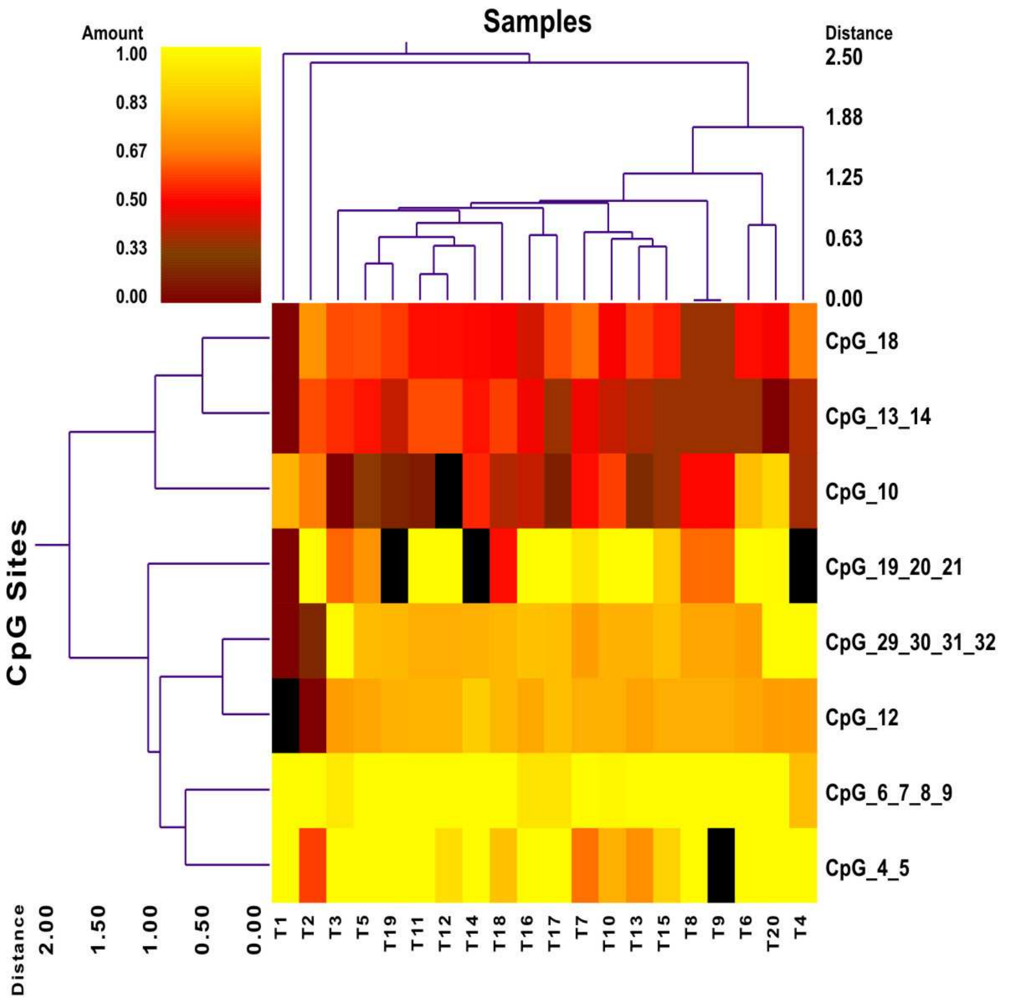

Breast Cancerous Serum

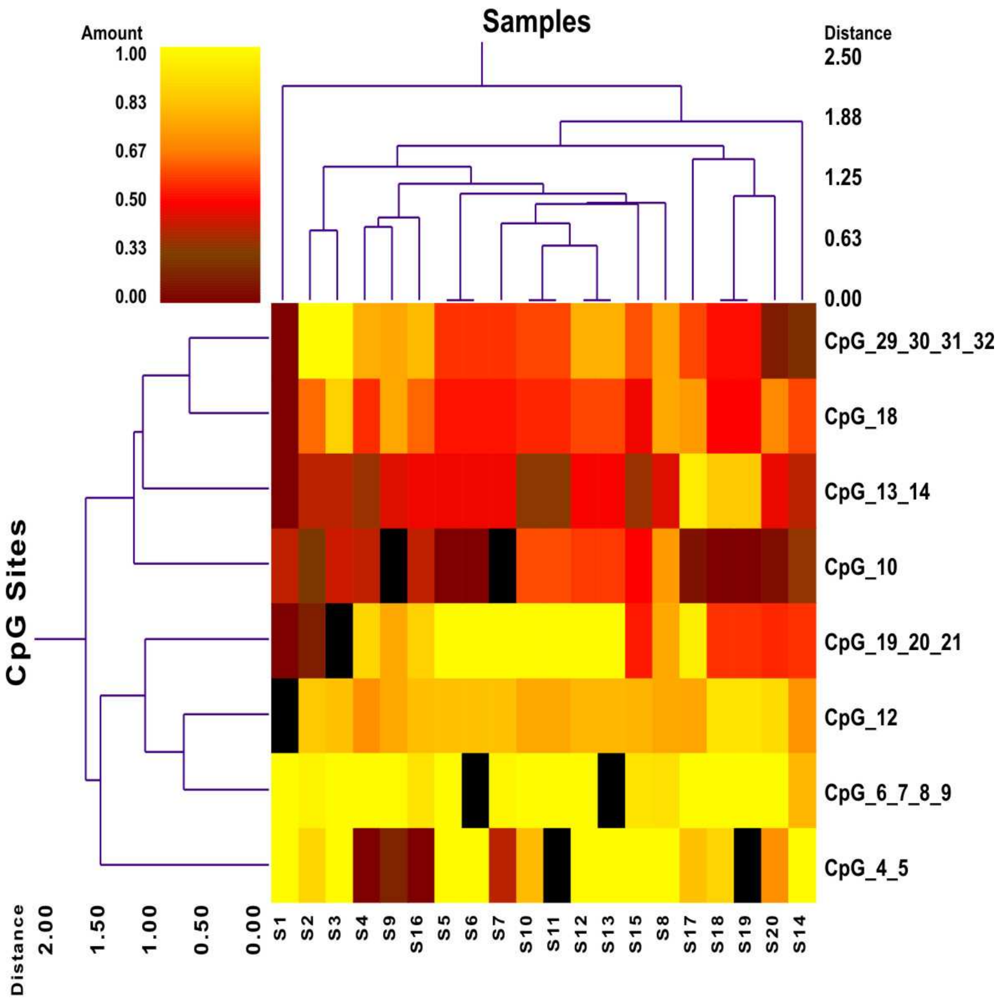

Adjacent Normal Tissue

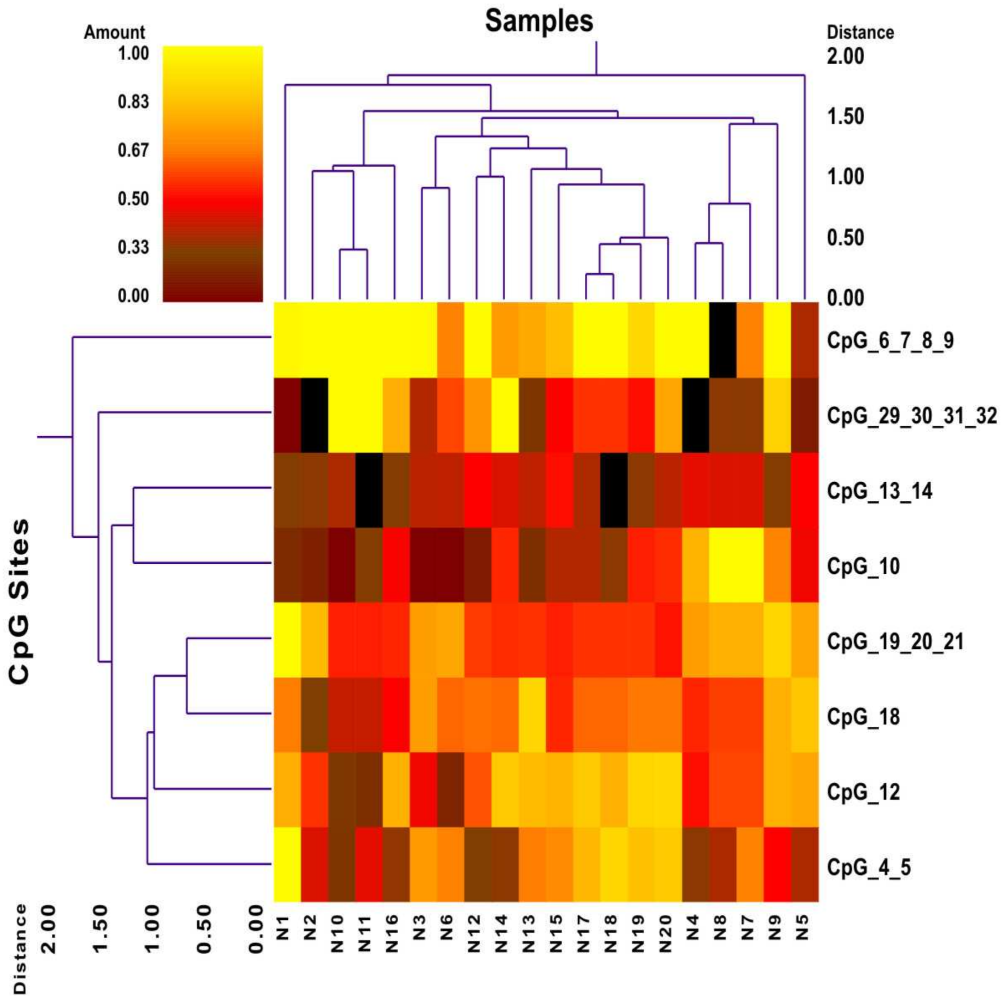

Double dendrogram of *BIN1* gene: Two-way hierarchical cluster analysis of 60 triple samples (breast cancerous tissue, matched normal tissue serum and samples) from 20 breast cancer patients. (Red clusters indicate 0% methylated, yellow clusters indicate 100% methylated, color gradient between red and yellow indicates methylation ranging from 0-100, and black clusters indicate not analyzed CpG sites).

BMP6 Gene

Gene ID	Alternate gene name	locus	Function	Methylation effect on breast cancer
654	bone morphogenetic protein 6	6p24-p23	The bone morphogenetic proteins (BMPs) are a family of secreted signaling molecules.	direct

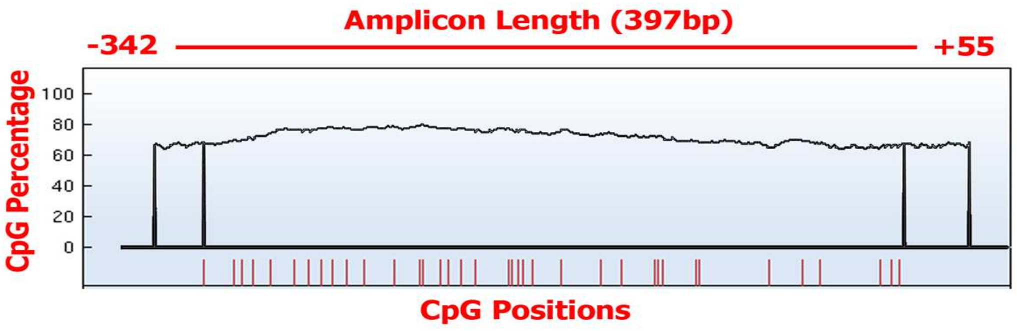

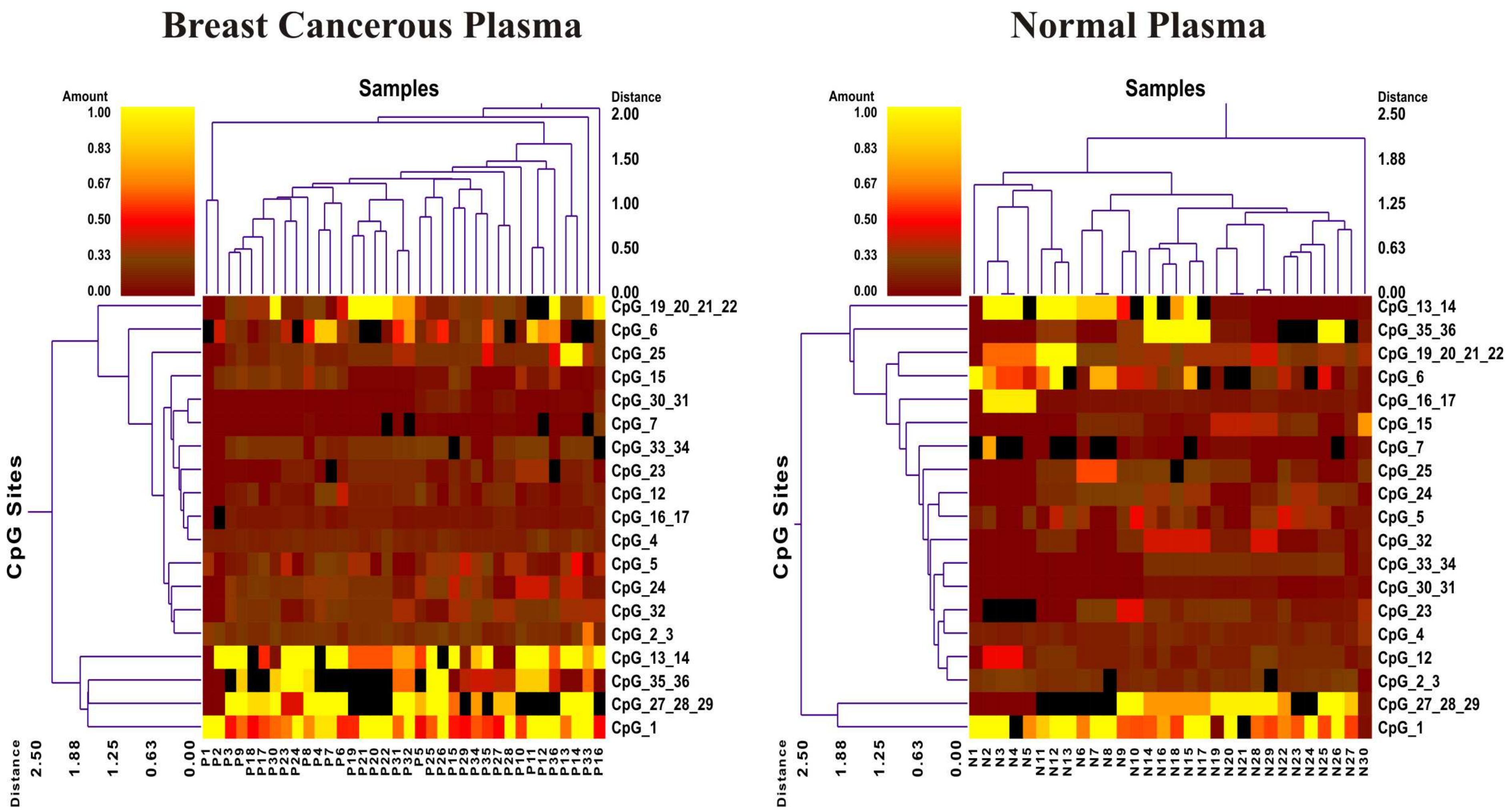

Double dendrogram of *BMP6* gene: Two-way hierarchical cluster analysis of 36 plasma samples from breast cancer patients and 30 plasma samples of normal subjects. (Red clusters indicate 0% methylated, yellow clusters indicate 100% methylated, color gradient between red and yellow indicates methylation ranging from 0-100, and black clusters indicate not analyzed CpG sites).

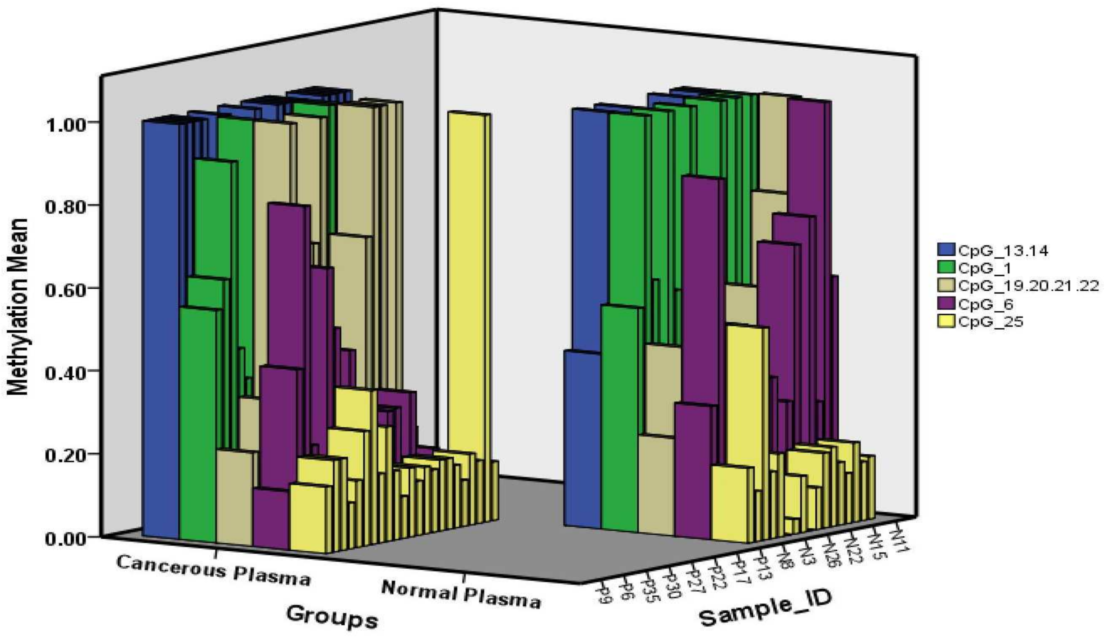

Comparison of informative CpG sites in two groups of plasma samples.

Breast Cancerous Tissue

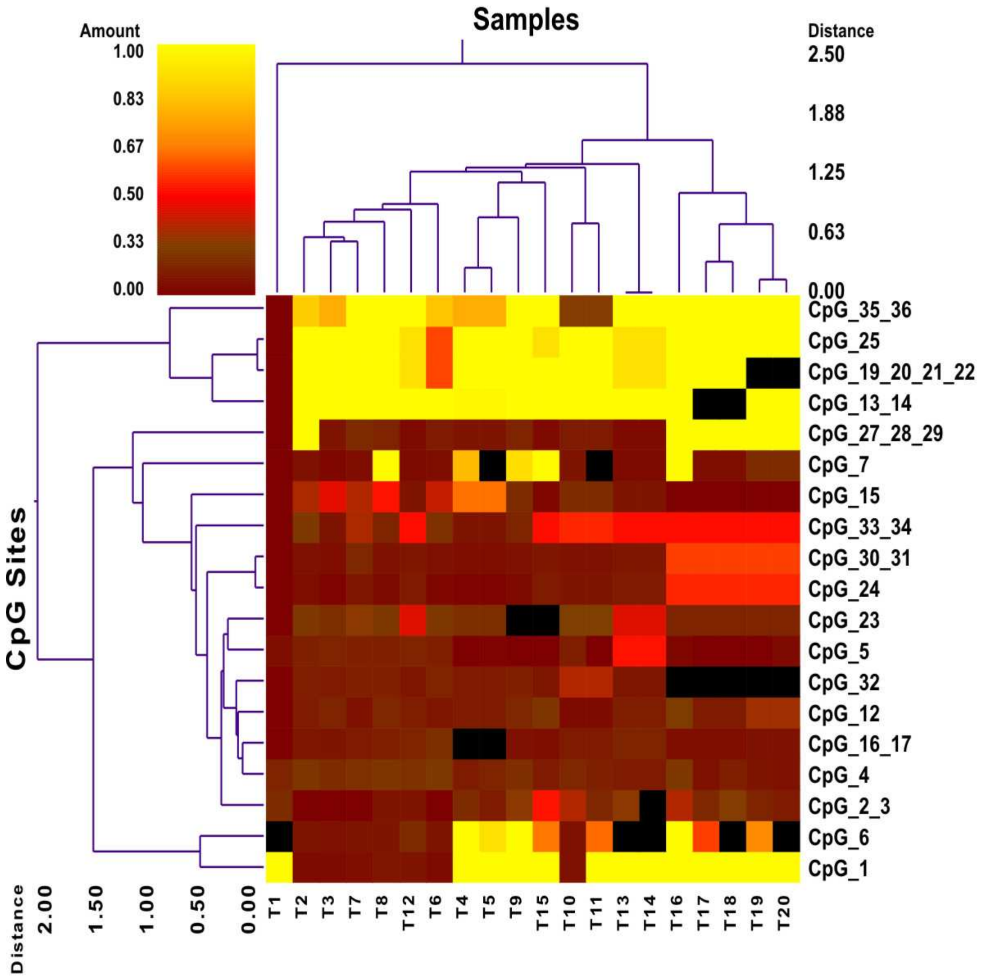

Breast Cancerous Serum

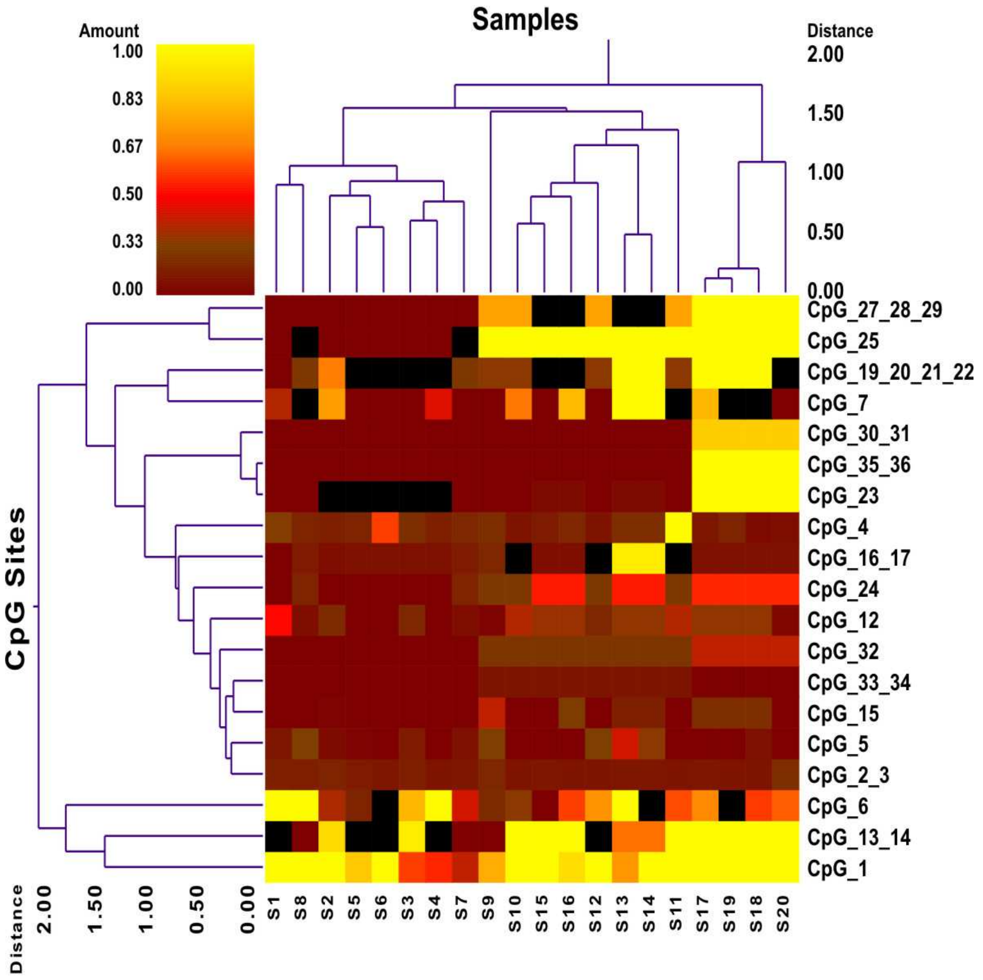

Adjacent Normal Tissue

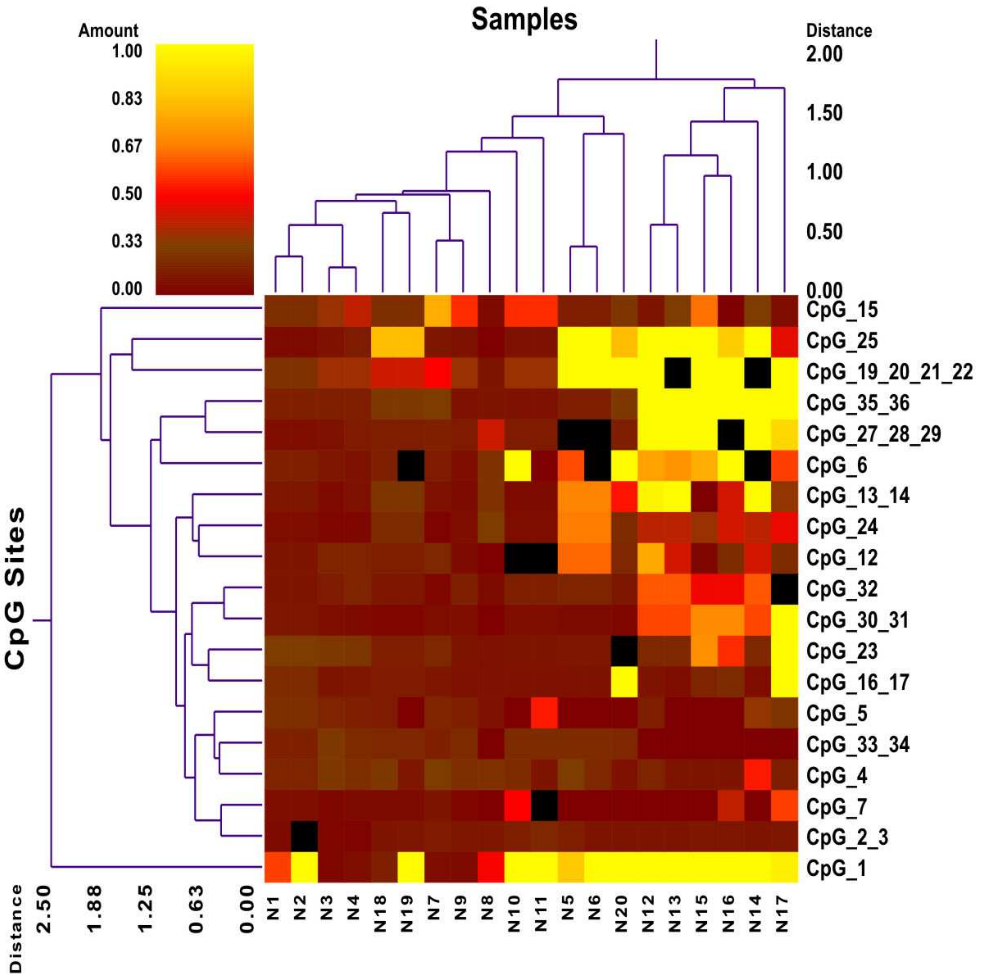

Double dendrogram of *BMP6* gene: Two-way hierarchical cluster analysis of 60 triple samples (breast cancerous tissue, matched normal tissue serum and samples) from 20 breast cancer patients. (Red clusters indicate 0% methylated, yellow clusters indicate 100% methylated, color gradient between red and yellow indicates methylation ranging from 0-100, and black clusters indicate not analyzed CpG sites).

BRCA1 Gene

Gene ID	Alternate gene name	locus	Function	Methylation effect on breast cancer
672	Breast Cancer type 1	17q21	Involved in DNA repair, recombination, checkpoint control of the cell cycle and transcription. Interacts with p53, STAT-factors, SRBC, etc.	direct

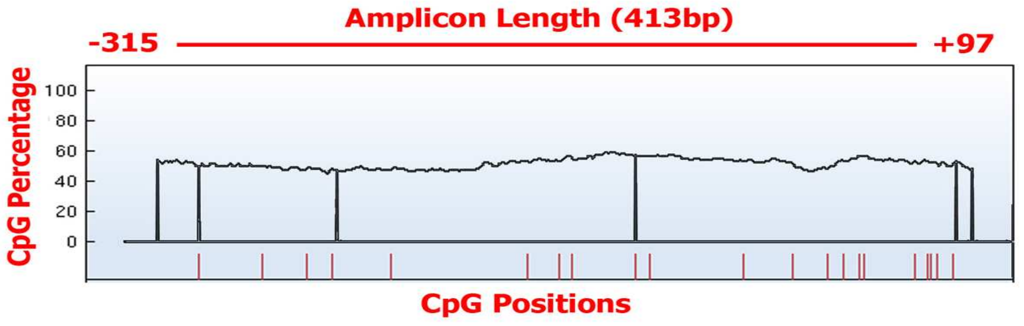

Breast Cancerous Plasma

Normal Plasma

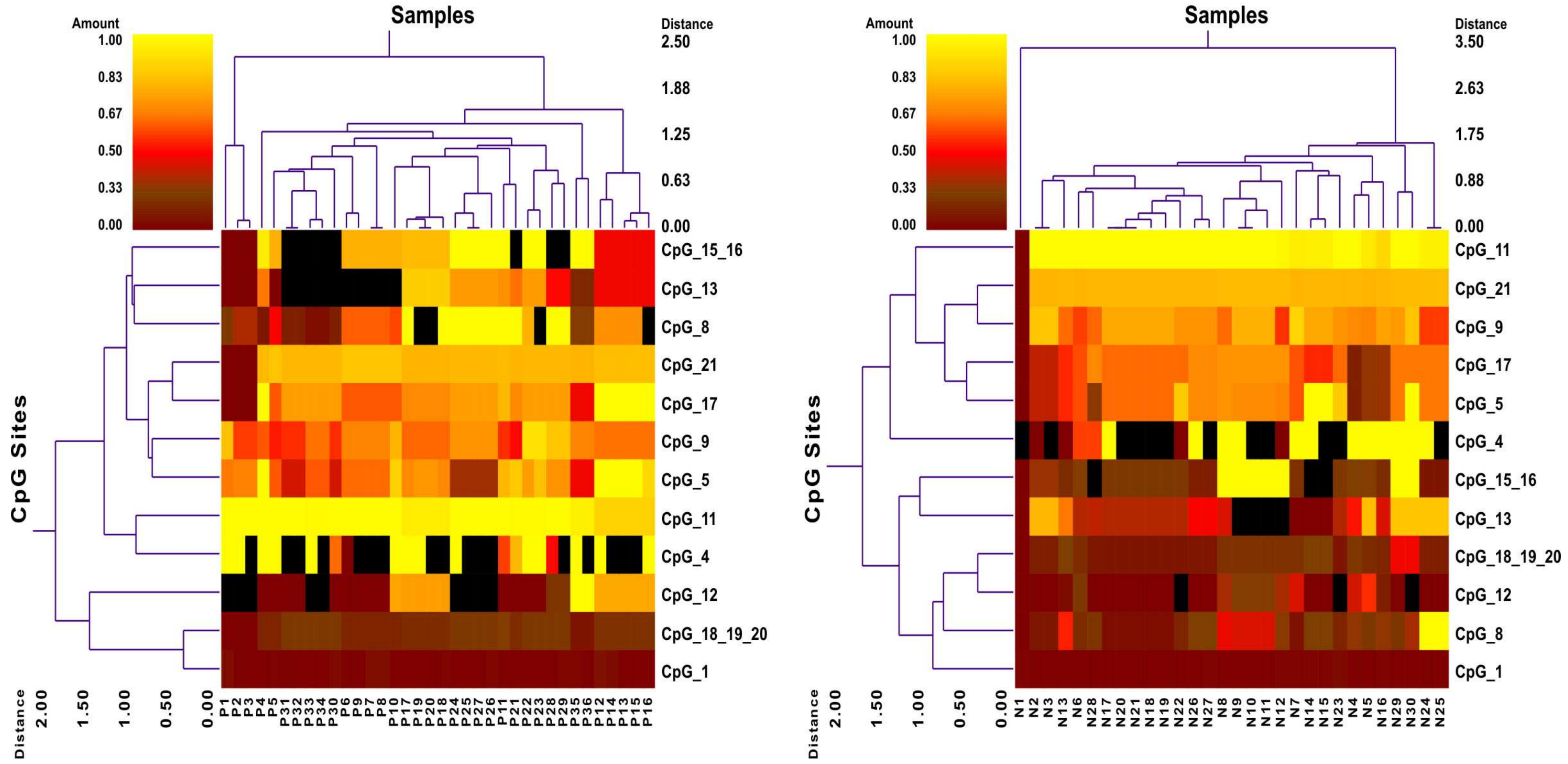

Double dendrogram of *BRCA1* gene: Two-way hierarchical cluster analysis of 36 plasma samples from breast cancer patients and 30 plasma samples of normal subjects. (Red clusters indicate 0% methylated, yellow clusters indicate 100% methylated, color gradient between red and yellow indicates methylation ranging from 0-100, and black clusters indicate not analyzed CpG sites).

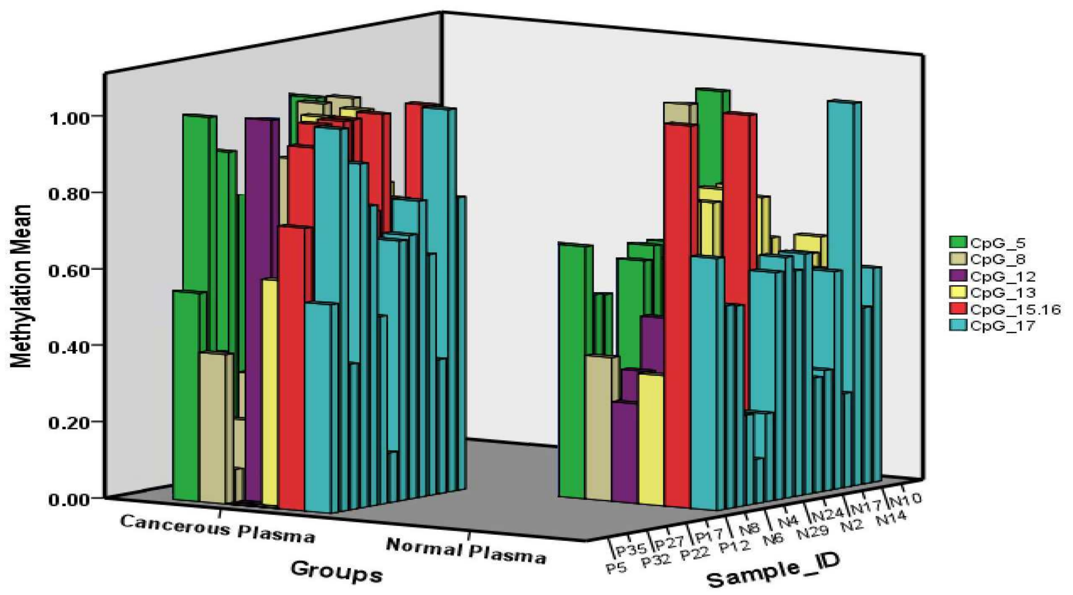

Comparison of informative CpG sites in two groups of plasma samples.

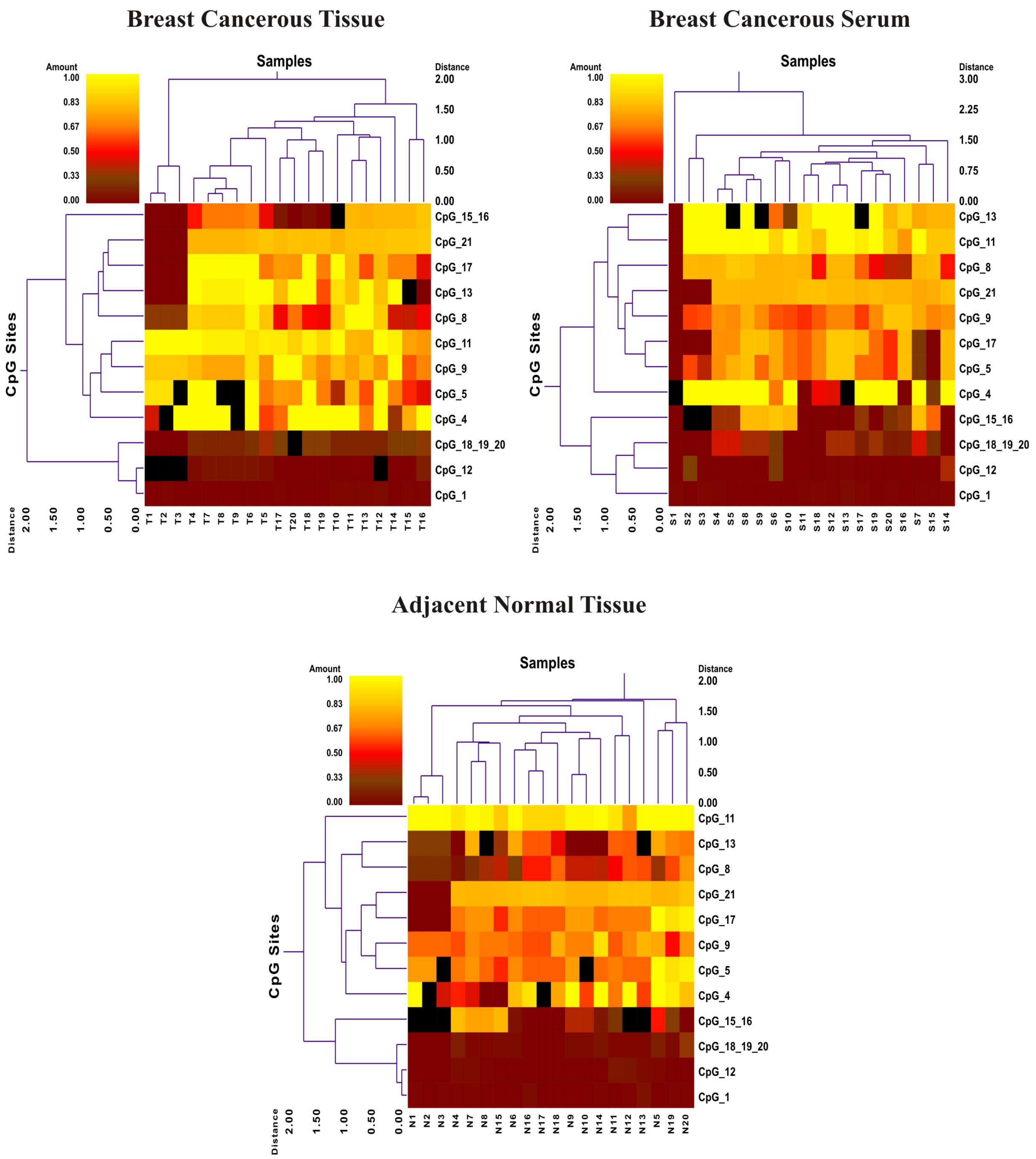

Double dendrogram of *BRCA1* gene: Two-way hierarchical cluster analysis of 60 triple samples (breast cancerous tissue, matched normal tissue serum and samples) from 20 breast cancer patients. (Red clusters indicate 0% methylated, yellow clusters indicate 100% methylated, color gradient between red and yellow indicates methylation ranging from 0-100, and black clusters indicate not analyzed CpG sites).

CST6 Gene

Gene ID	Alternate gene name	locus	Function	Methylation effect on breast cancer
1474	Cystatin E/M	11q13	This gene encodes a cystatin from type 2 family, which is down-regulated in metastatic breast tumour cells as compared to primary tumour cells. Loss of expression is likely associated with the progression of a primary tumour to a metastatic phenotype.	direct

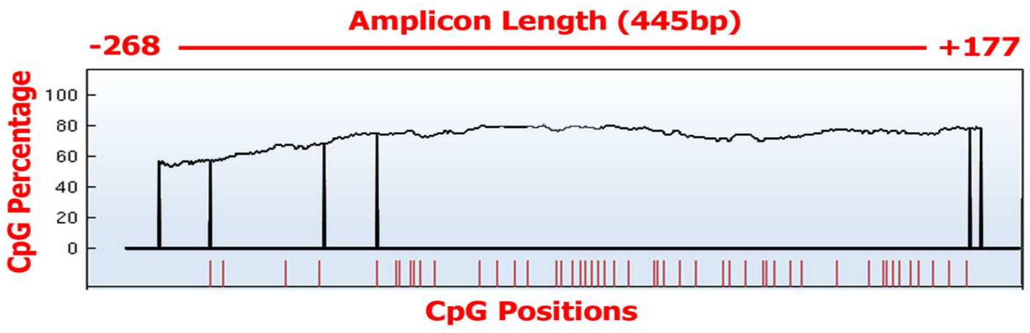

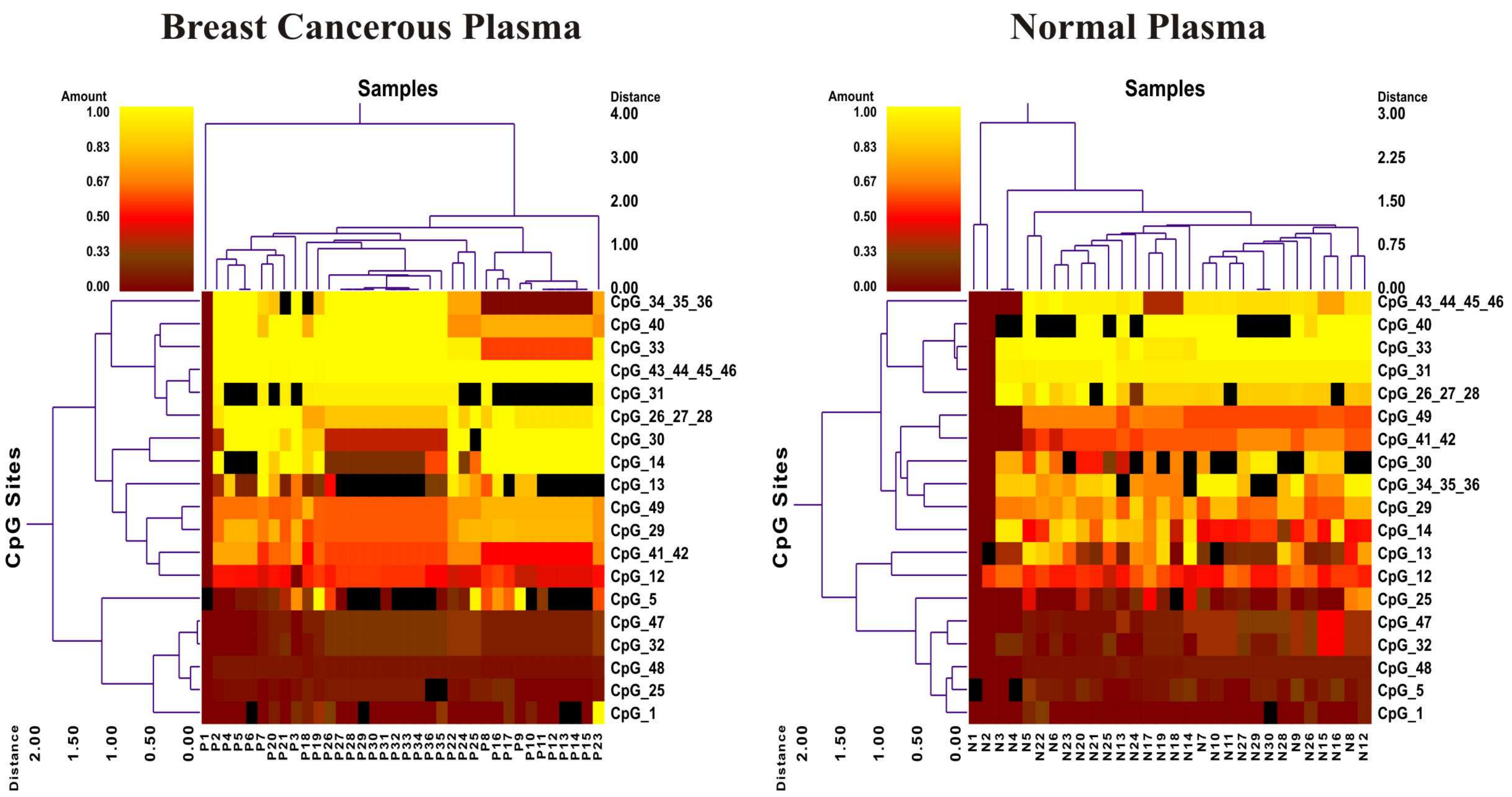

Double dendrogram of *CST6* gene: Two-way hierarchical cluster analysis of 36 plasma samples from breast cancer patients and 30 plasma samples of normal subjects. (Red clusters indicate 0% methylated, yellow clusters indicate 100% methylated, color gradient between red and yellow indicates methylation ranging from 0-100, and black clusters indicate not analyzed CpG sites).

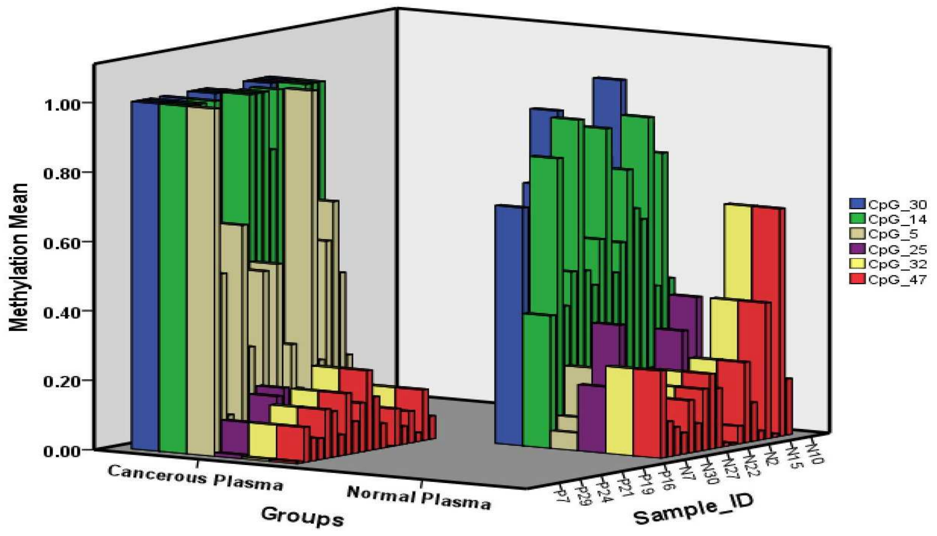

Comparison of informative CpG sites in two groups of plasma samples.

Breast Cancerous Tissue

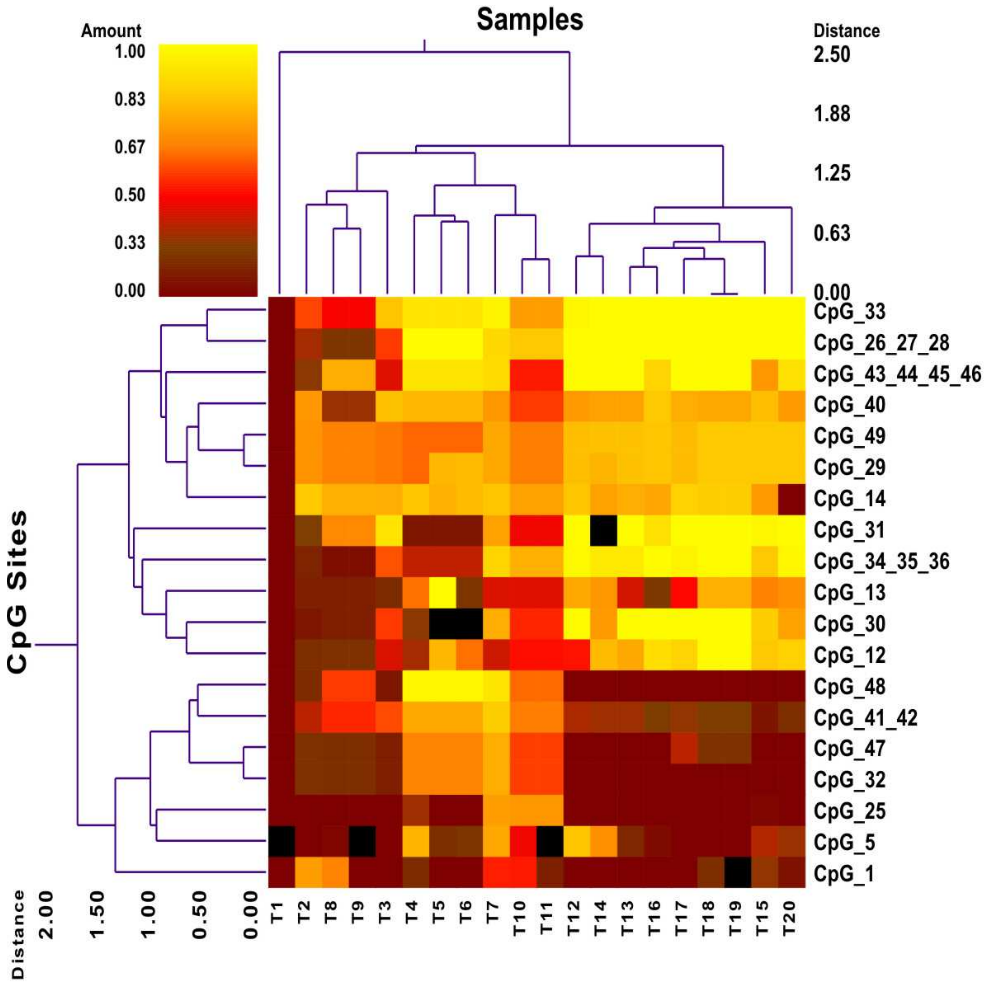

Breast Cancerous Serum

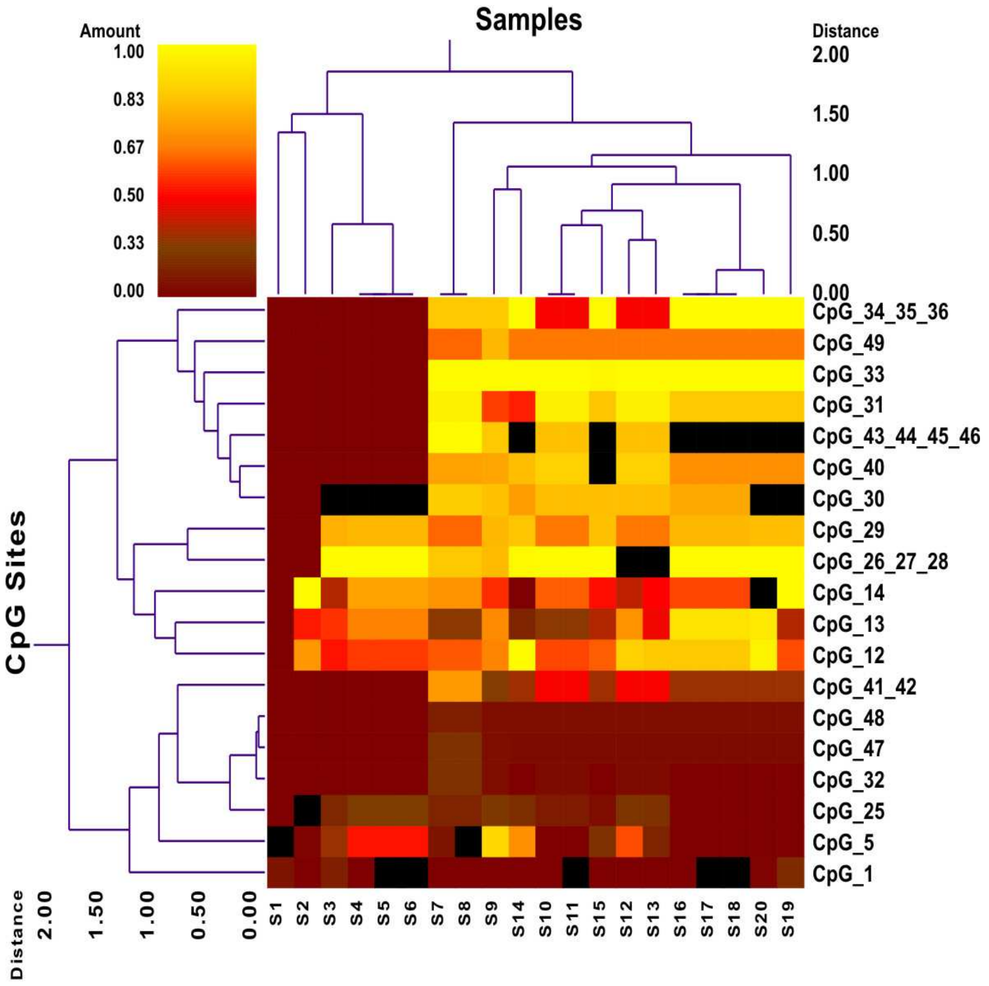

Adjacent Normal Tissue

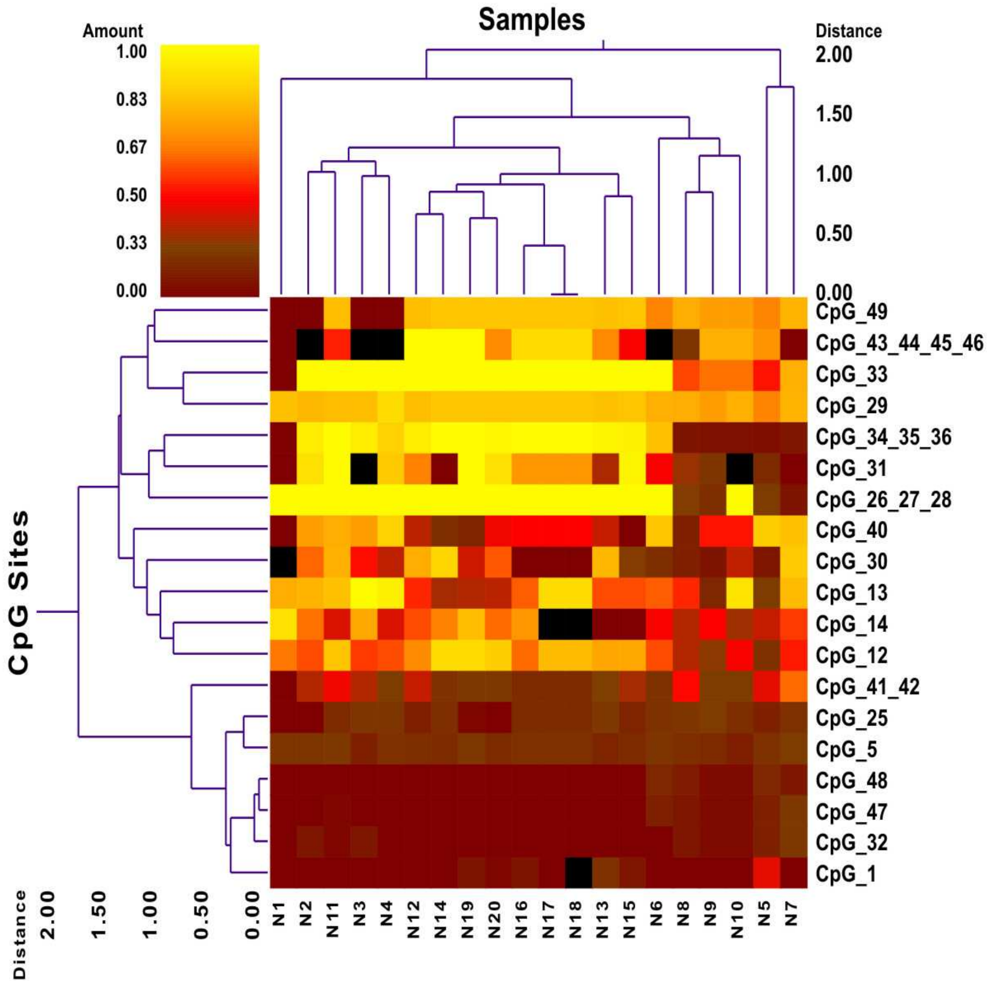

Double dendrogram of *CST6* gene: Two-way hierarchical cluster analysis of 60 triple samples (breast cancerous tissue, matched normal tissue serum and samples) from 20 breast cancer patients. (Red clusters indicate 0% methylated, yellow clusters indicate 100% methylated, color gradient between red and yellow indicates methylation ranging from 0-100, and black clusters indicate not analyzed CpG sites).

ESR-b Gene

Gene ID	Alternate gene name	locus	Function	Methylation effect on breast cancer
2100	estrogen receptor 2 (ER beta)	14q23.2	Regulation of cell proliferation, predictor of endocrine therapy	direct

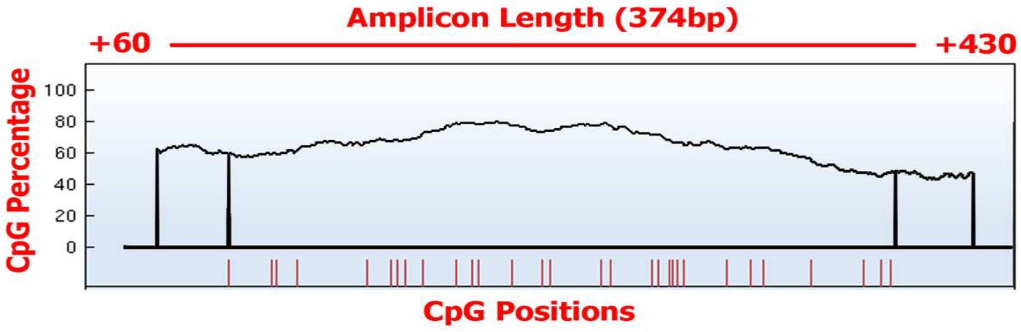

Breast Cancerous Plasma   Normal Plasma

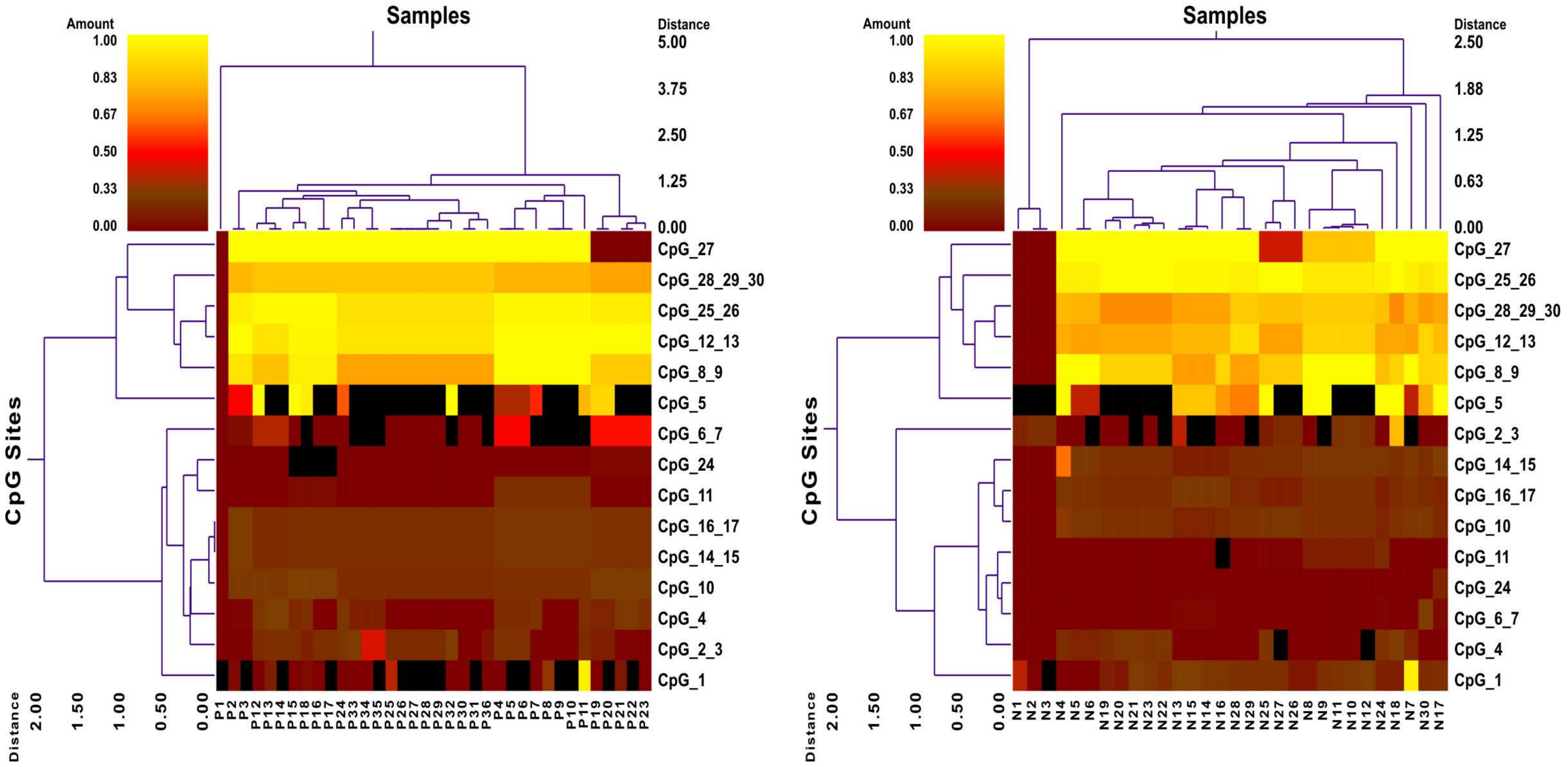

Double dendrogram of *ESR-b* gene: Two-way hierarchical cluster analysis of 36 plasma samples from breast cancer patients and 30 plasma samples of normal subjects. (Red clusters indicate 0% methylated, yellow clusters indicate 100% methylated, color gradient between red and yellow indicates methylation ranging from 0-100, and black clusters indicate not analyzed CpG sites).

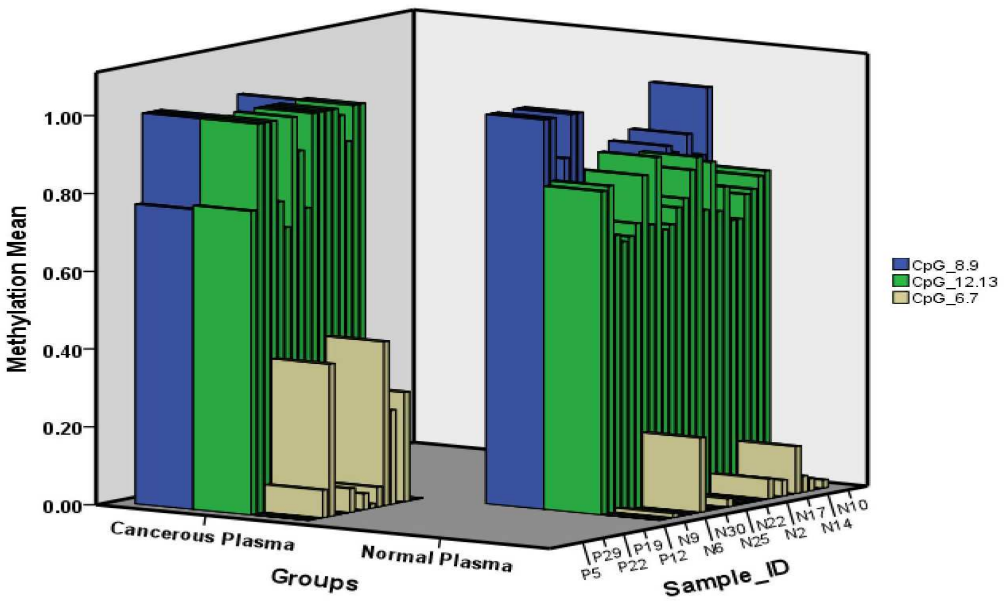

Comparison of informative CpG sites in two groups of plasma samples.

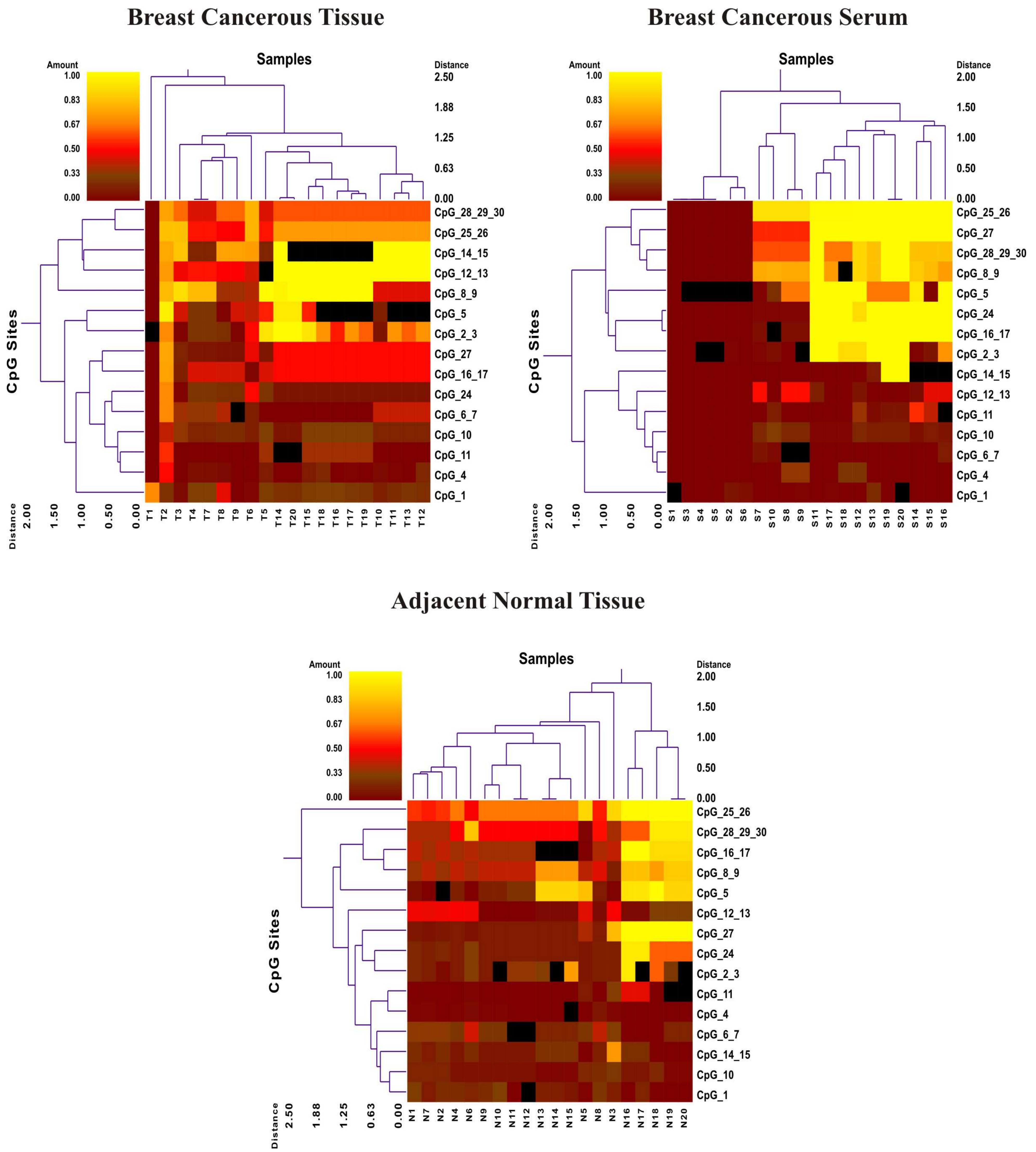

Double dendrogram of *ESR-b* gene: Two-way hierarchical cluster analysis of 60 triple samples (breast cancerous tissue, matched normal tissue serum and samples) from 20 breast cancer patients. (Red clusters indicate 0% methylated, yellow clusters indicate 100% methylated, color gradient between red and yellow indicates methylation ranging from 0-100, and black clusters indicate not analyzed CpG sites).

GSTP1 Gene

Gene ID	Alternate gene name	locus	Function	Methylation effect on breast cancer
2950	Glutathione S-transferase P1	11q13	Carcinogen detoxification. GSTP1 is a polymorphic gene encoding active, functionally different GSTP1 variant proteins that are thought to function in xenobiotic metabolism and play a role in susceptibility to cancer, and other diseases.	direct

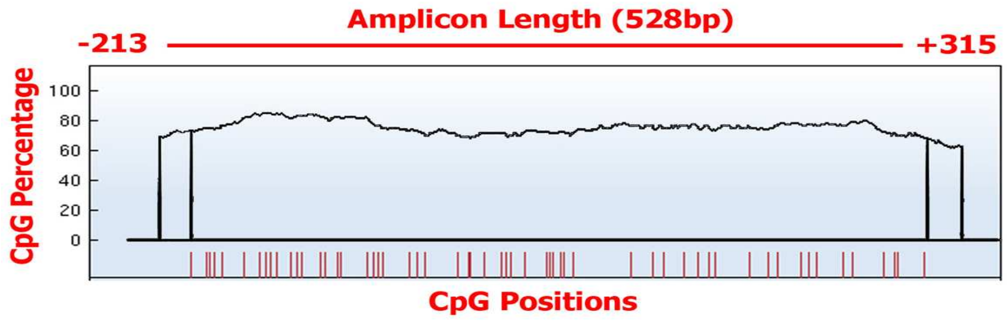

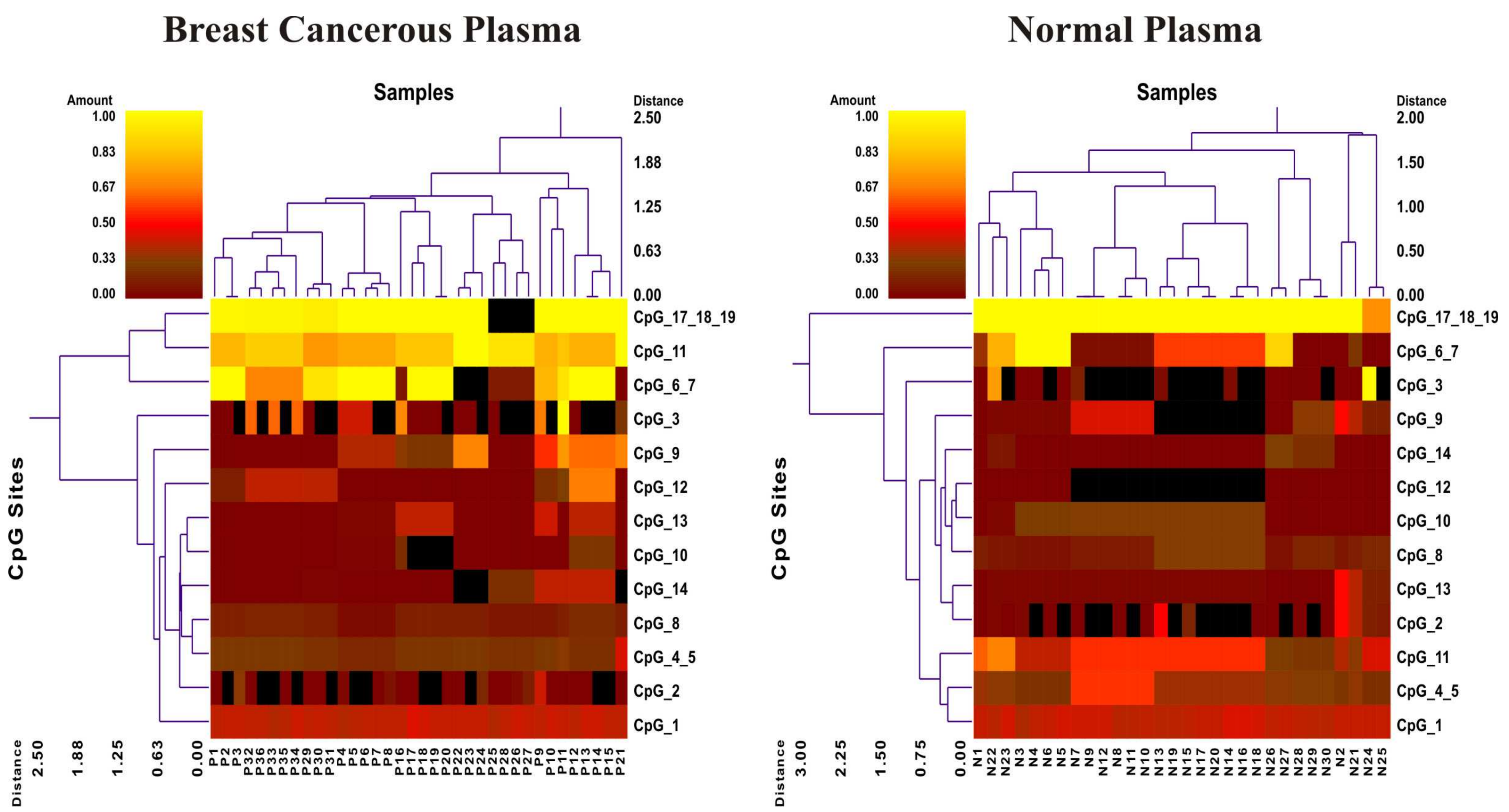

Double dendrogram of *GSTP1* gene: Two-way hierarchical cluster analysis of 36 plasma samples from breast cancer patients and 30 plasma samples of normal subjects. (Red clusters indicate 0% methylated, yellow clusters indicate 100% methylated, color gradient between red and yellow indicates methylation ranging from 0-100, and black clusters indicate not analyzed CpG sites).

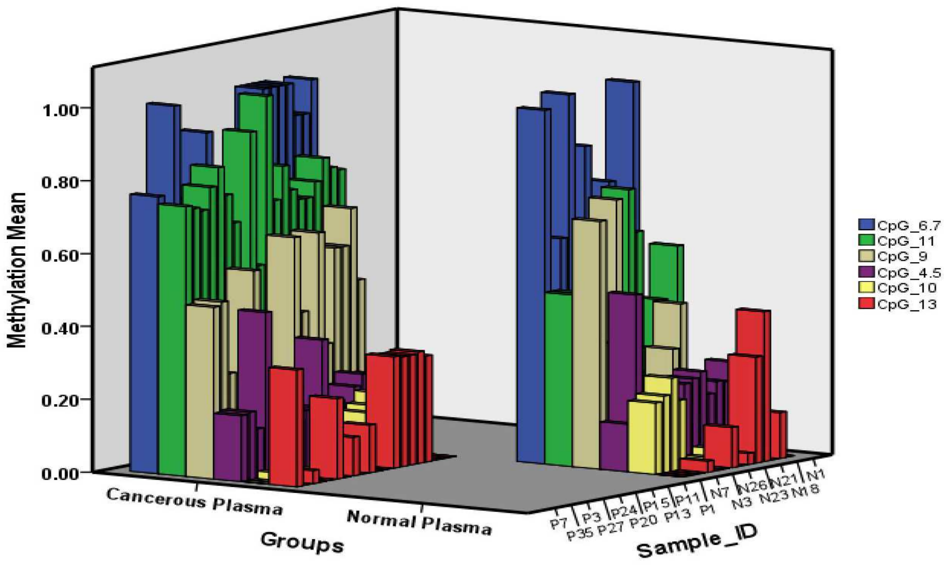

Comparison of informative CpG sites in two groups of plasma samples.

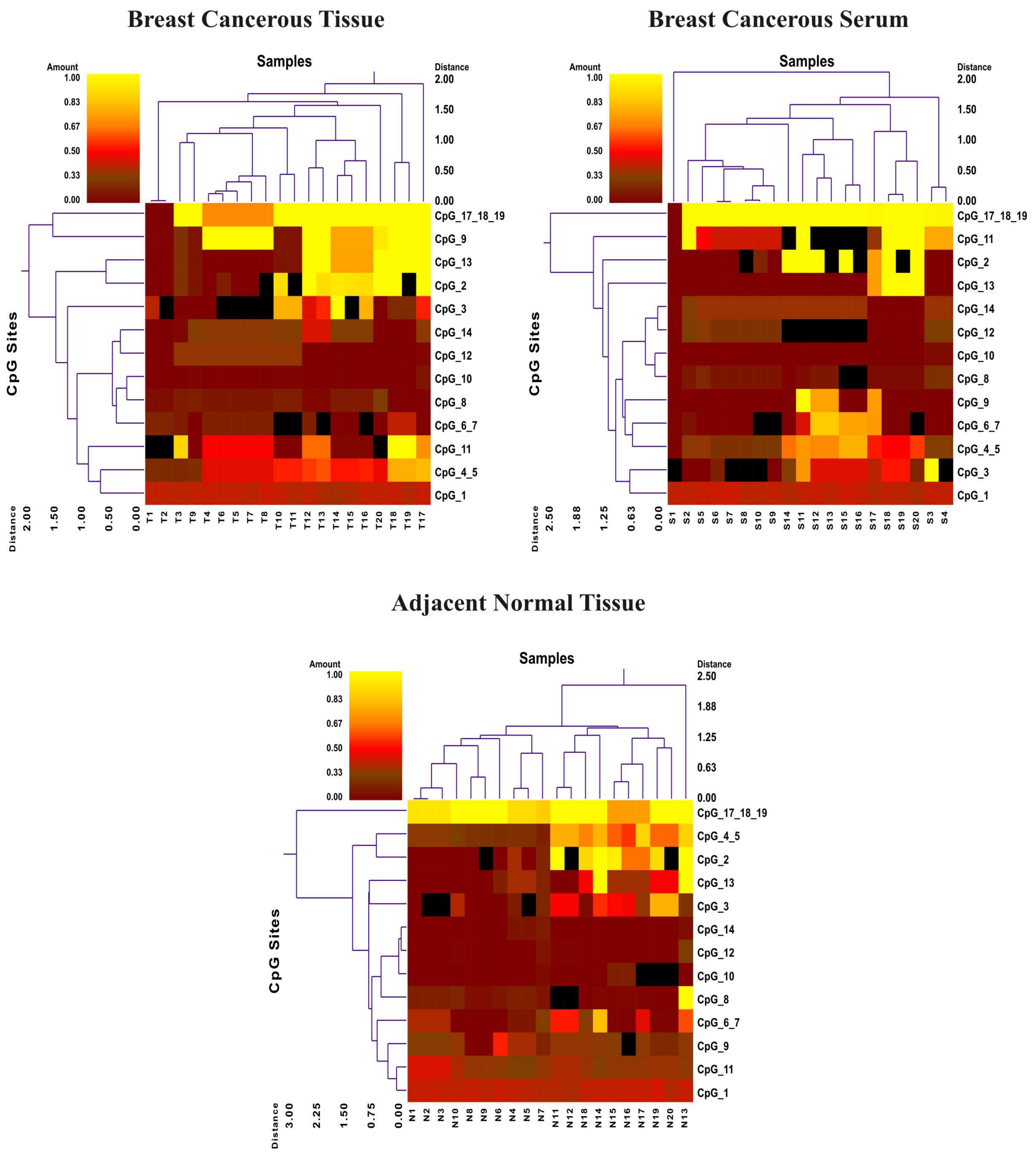

Double dendrogram of *GSTP1* gene: Two-way hierarchical cluster analysis of 60 triple samples (breast cancerous tissue, matched normal tissue serum and samples) from 20 breast cancer patients. (Red clusters indicate 0% methylated, yellow clusters indicate 100% methylated, color gradient between red and yellow indicates methylation ranging from 0-100, and black clusters indicate not analyzed CpG sites).

P16 (CDKN2A) Gene

Gene ID	Alternate gene name	locus	Function	Methylation effect on breast cancer
1029	Cyclin-dependent kinase inhibitor 2A (melanoma, p16, inhibits CDK4)	9p21	Cell cycle regulation, involved in senescence.	direct

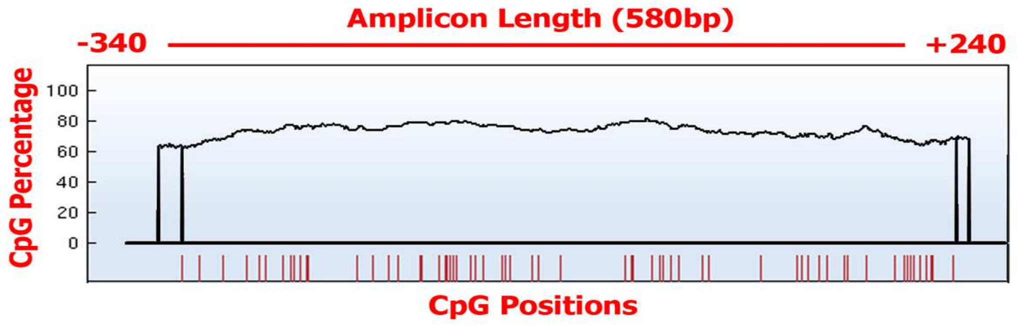

Breast Cancerous Plasma | **Normal Plasma**

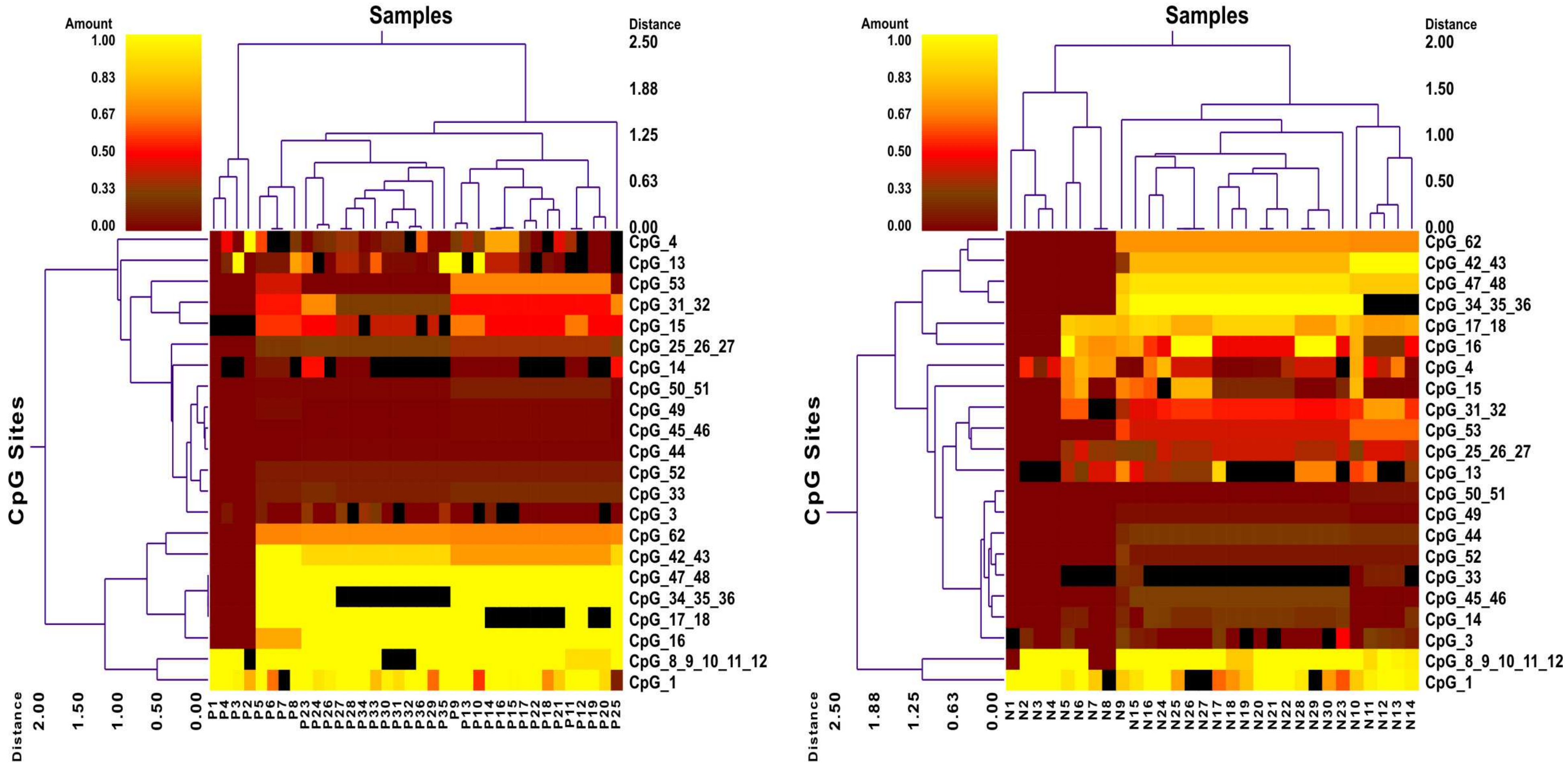

Double dendrogram of *P16* gene: Two-way hierarchical cluster analysis of 36 plasma samples from breast cancer patients and 30 plasma samples of normal subjects. (Red clusters indicate 0% methylated, yellow clusters indicate 100% methylated, color gradient between red and yellow indicates methylation ranging from 0-100, and black clusters indicate not analyzed CpG sites).

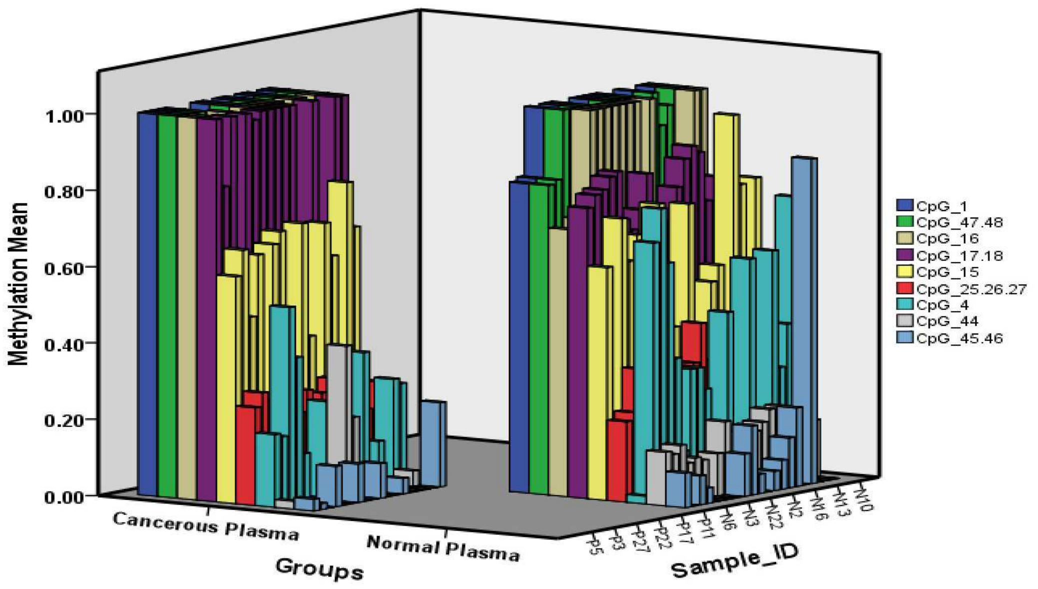

Comparison of informative CpG sites in two groups of plasma samples.

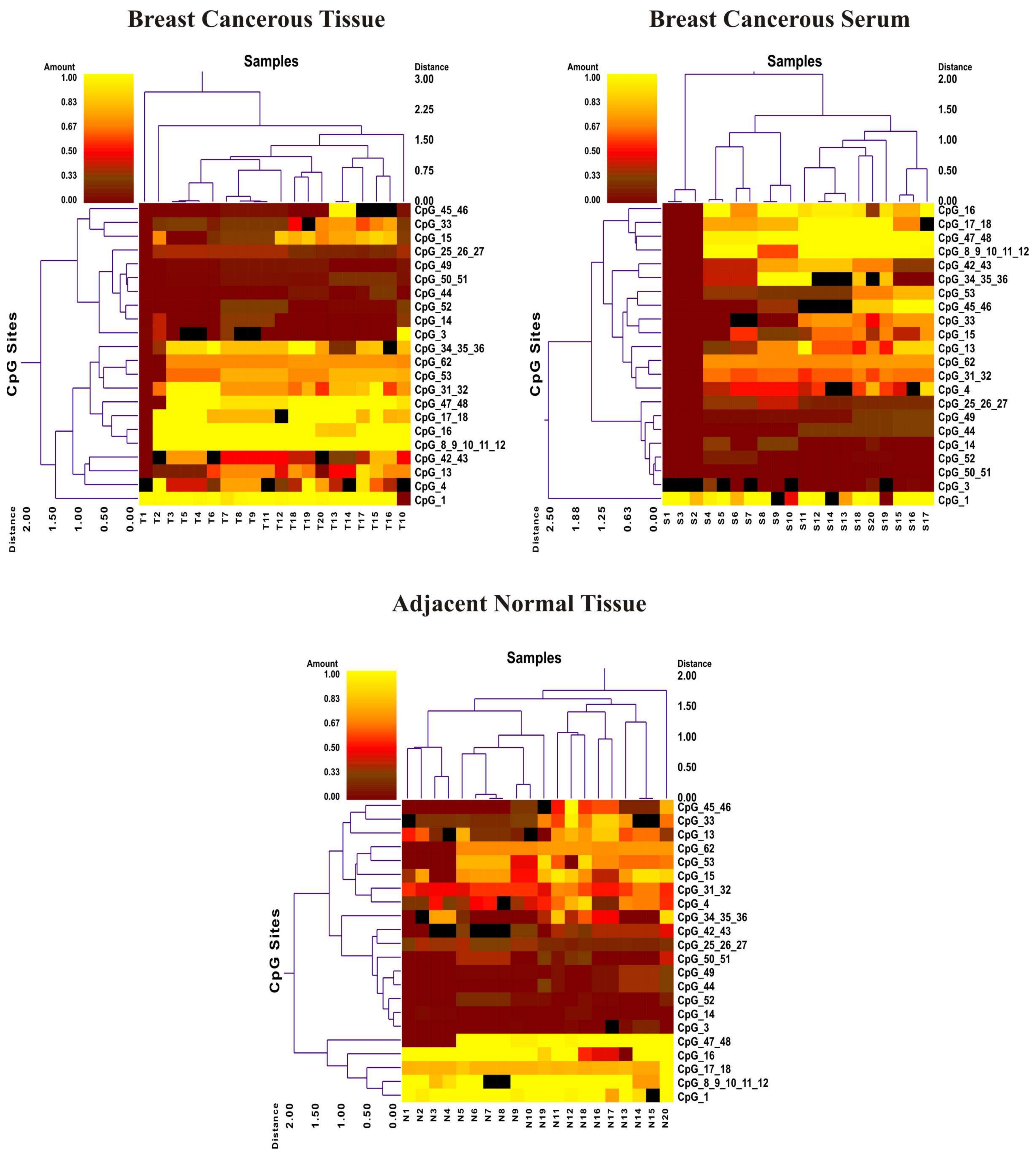

Double dendrogram of *P16* gene: Two-way hierarchical cluster analysis of 60 triple samples (breast cancerous tissue, matched normal tissue serum and samples) from 20 breast cancer patients. (Red clusters indicate 0% methylated, yellow clusters indicate 100% methylated, color gradient between red and yellow indicates methylation ranging from 0-100, and black clusters indicate not analyzed CpG sites).

P21 (CDKN1A) Gene

Gene ID	Alternate gene name	locus	Function	Methylation effect on breast cancer
1026	Cyclin-dependent kinase inhibitor 1A (p21, Cip1)	6p21.2	Encodes protein that binds to and inhibits the activity of cyclin-CDK2 or -CDK4 complexes, and thus functions as a regulator of cell cycle progression at G1. This protein plays a regulatory role in S phase DNA replication and DNA damage repair.	direct

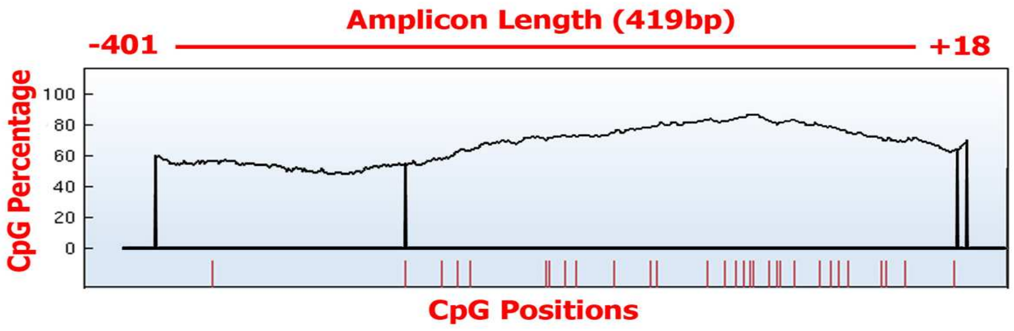

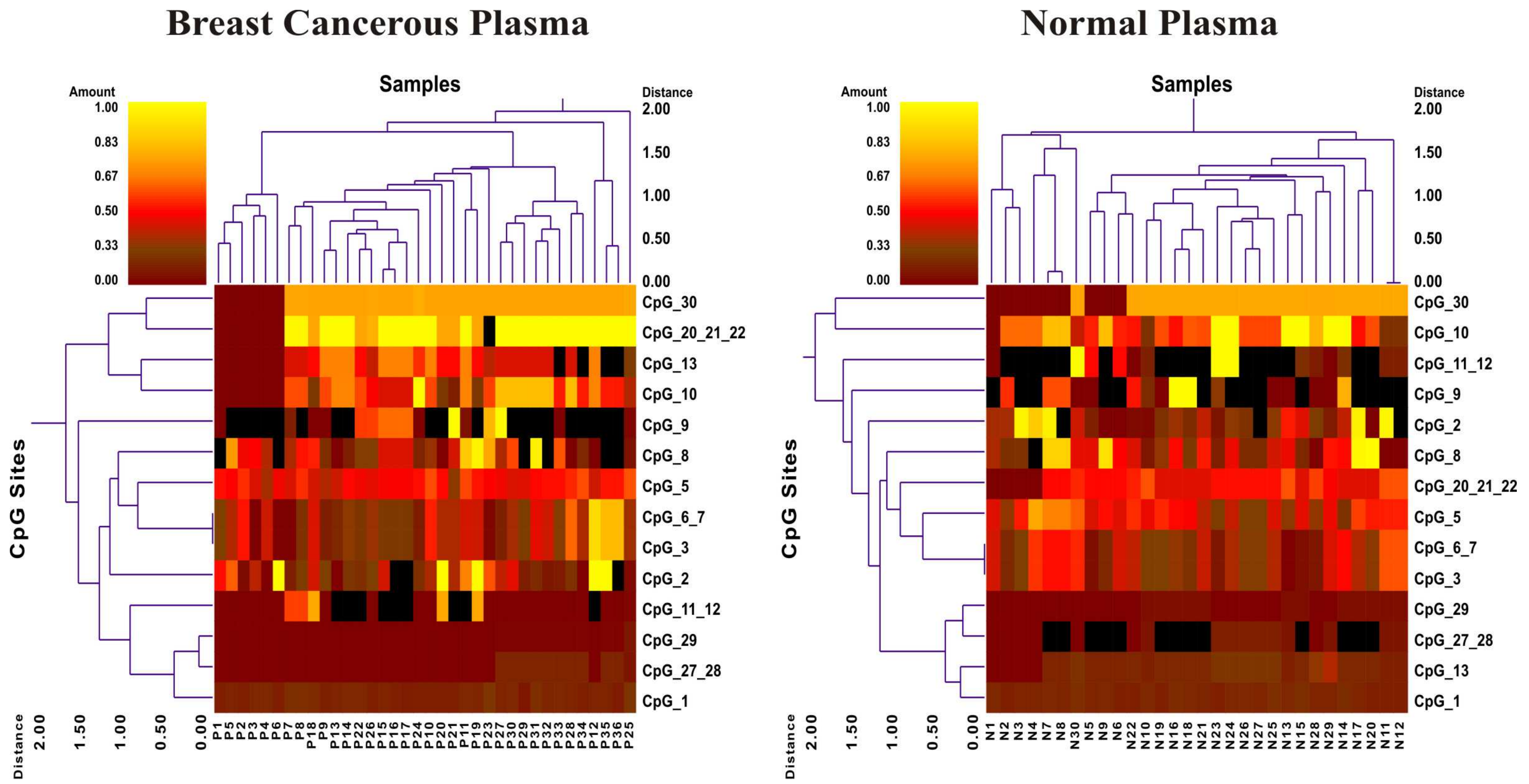

Double dendrogram of *P21* gene: Two-way hierarchical cluster analysis of 36 plasma samples from breast cancer patients and 30 plasma samples of normal subjects. (Red clusters indicate 0% methylated, yellow clusters indicate 100% methylated, color gradient between red and yellow indicates methylation ranging from 0-100, and black clusters indicate not analyzed CpG sites).

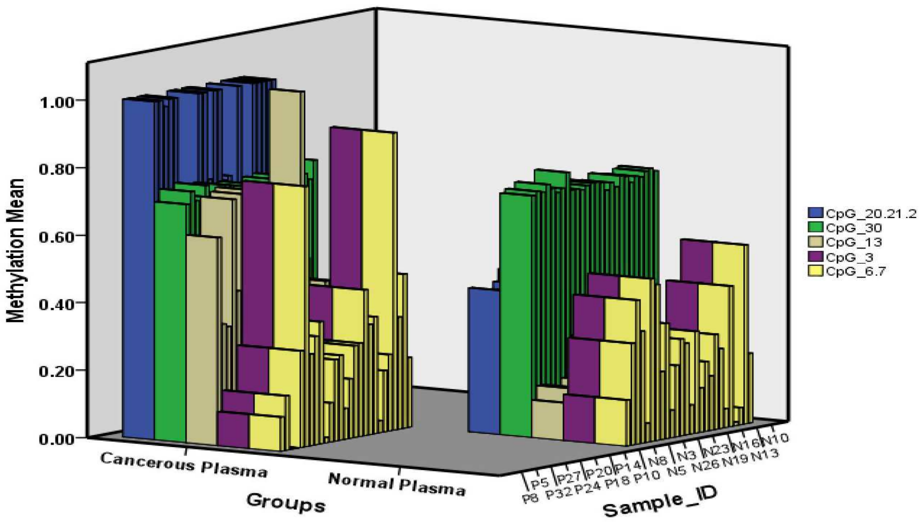

Comparison of informative CpG sites in two groups of plasma samples.

Breast Cancerous Tissue

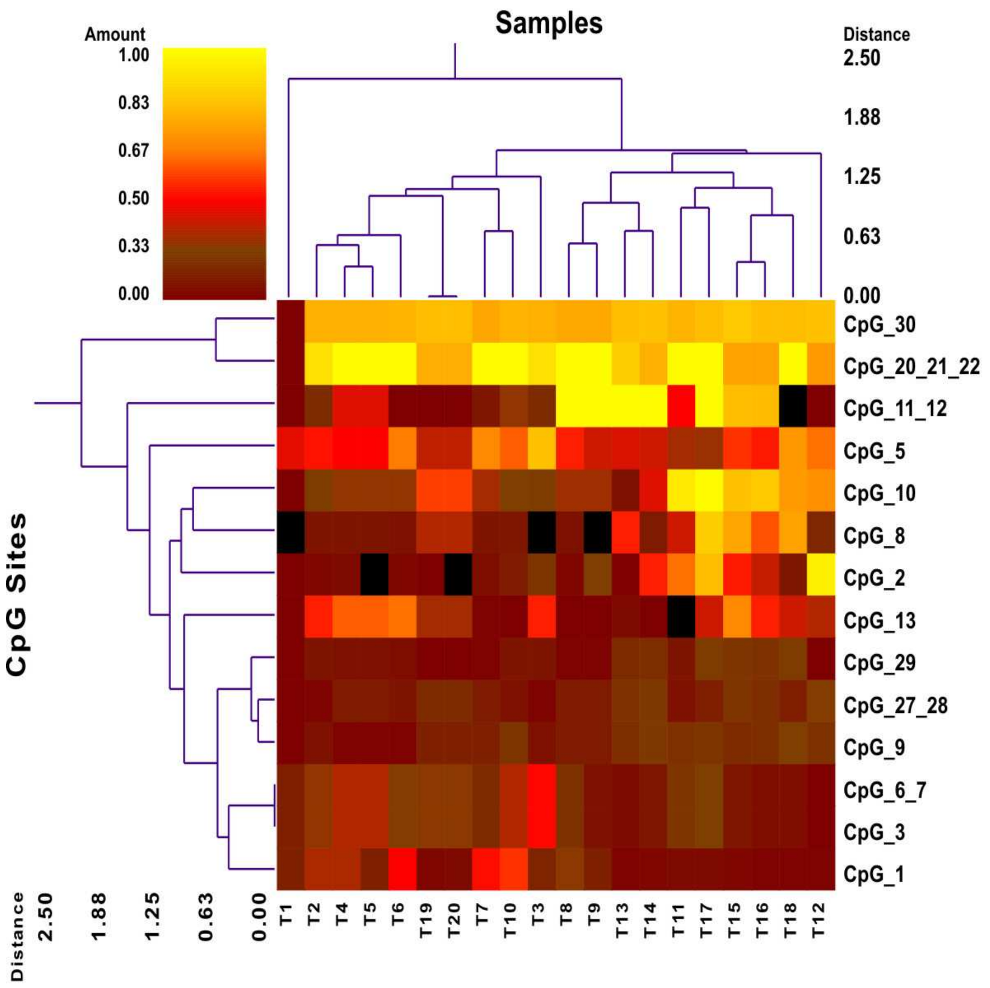

Breast Cancerous Serum

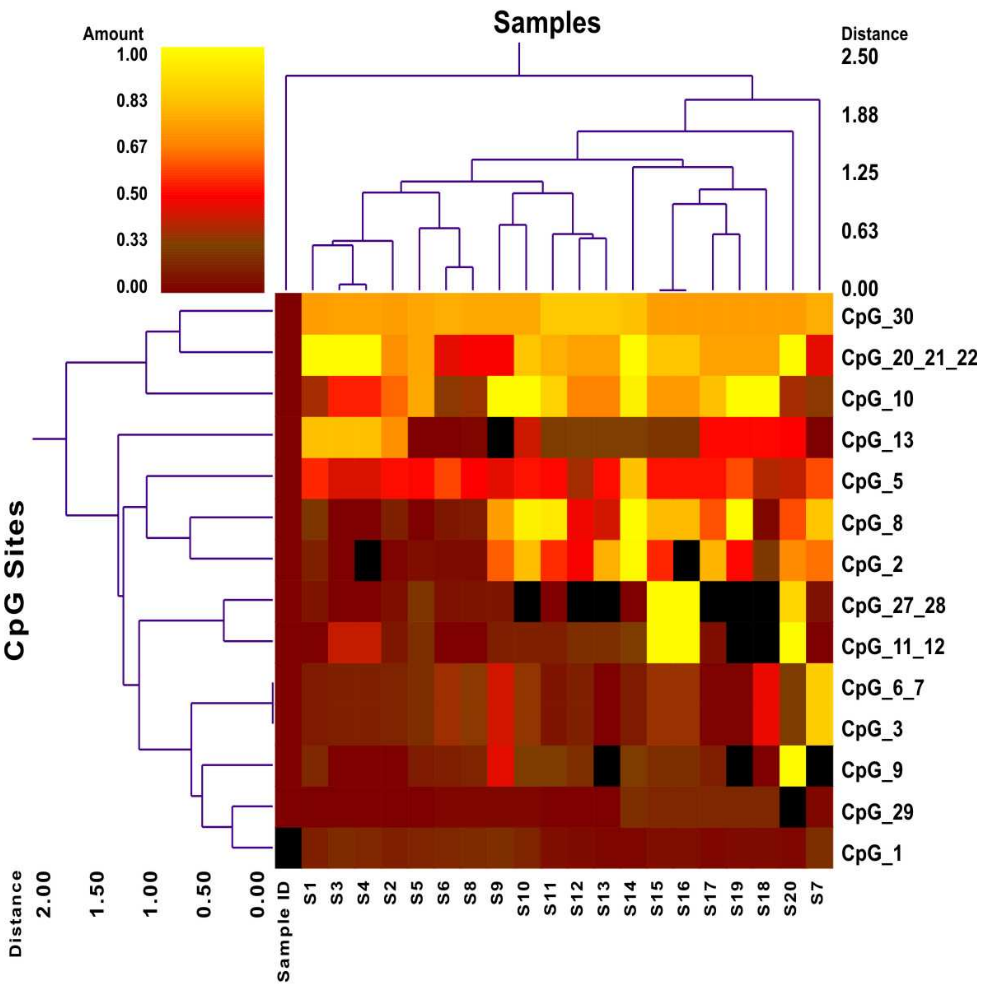

Adjacent Normal Tissue

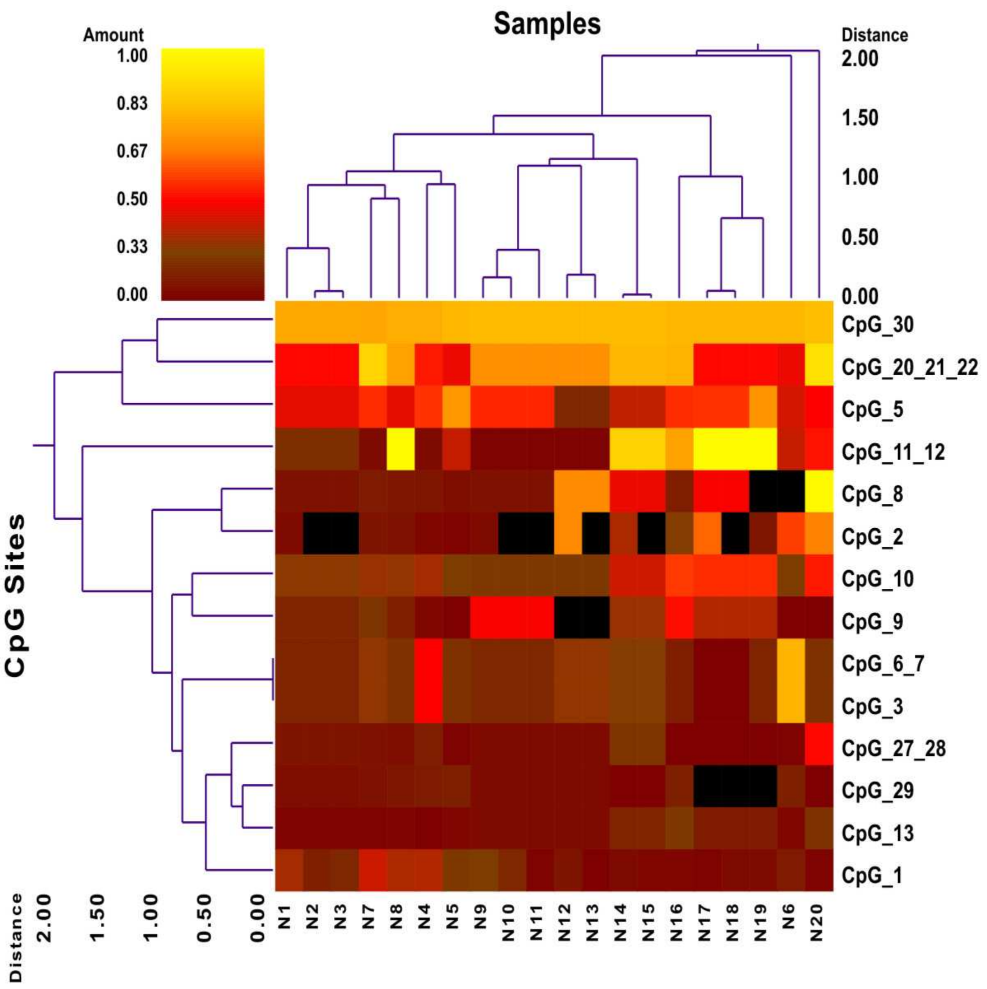

Double dendrogram of *P21* gene: Two-way hierarchical cluster analysis of 60 triple samples (breast cancerous tissue, matched normal tissue serum and samples) from 20 breast cancer patients. (Red clusters indicate 0% methylated, yellow clusters indicate 100% methylated, color gradient between red and yellow indicates methylation ranging from 0-100, and black clusters indicate not analyzed CpG sites).

TIMP3 Gene

Gene ID	Alternate gene name	locus	Function	Methylation effect on breast cancer
7078	Tissue inhibitor of metalloproteinase-3	22q12.3	Suppresses tumour growth, angiogenesis, invasion and metastasis	direct

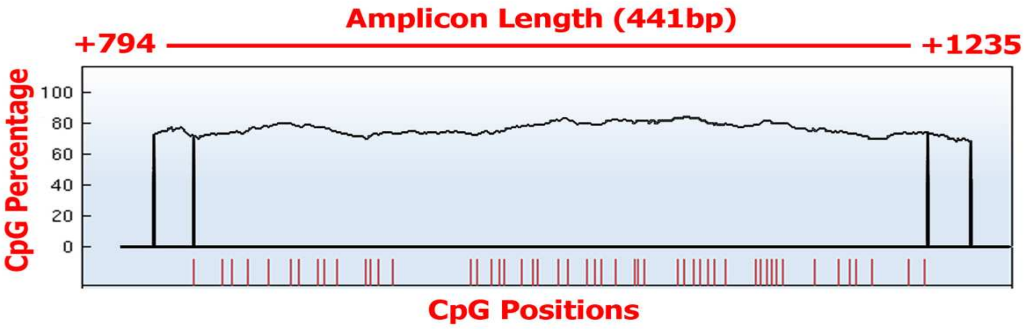

Breast Cancerous Plasma

Normal Plasma

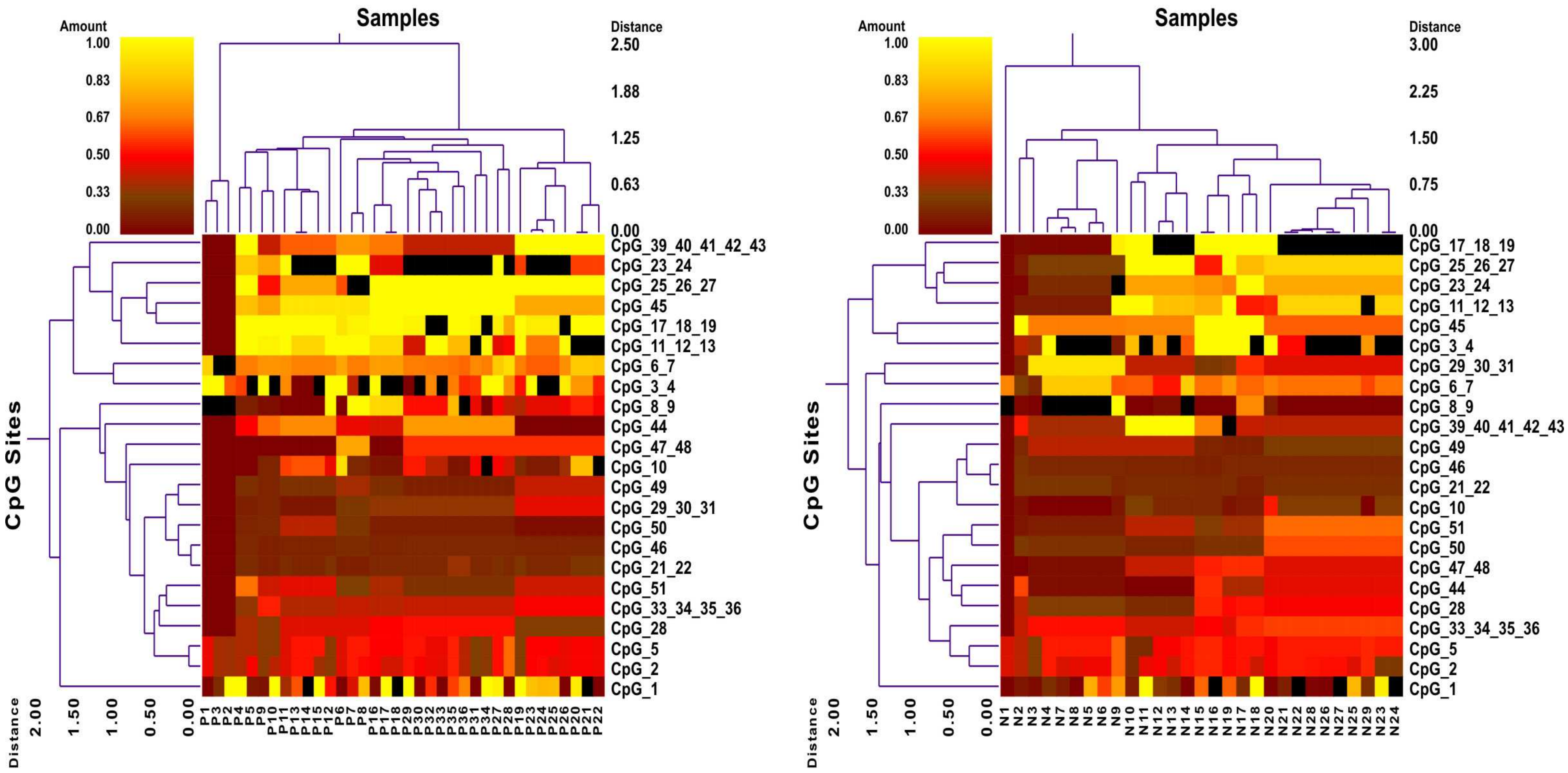

Double dendrogram of *TIMP3* gene: Two-way hierarchical cluster analysis of 36 plasma samples from breast cancer patients and 30 plasma samples of normal subjects. (Red clusters indicate 0% methylated, yellow clusters indicate 100% methylated, color gradient between red and yellow indicates methylation ranging from 0-100, and black clusters indicate not analyzed CpG sites).

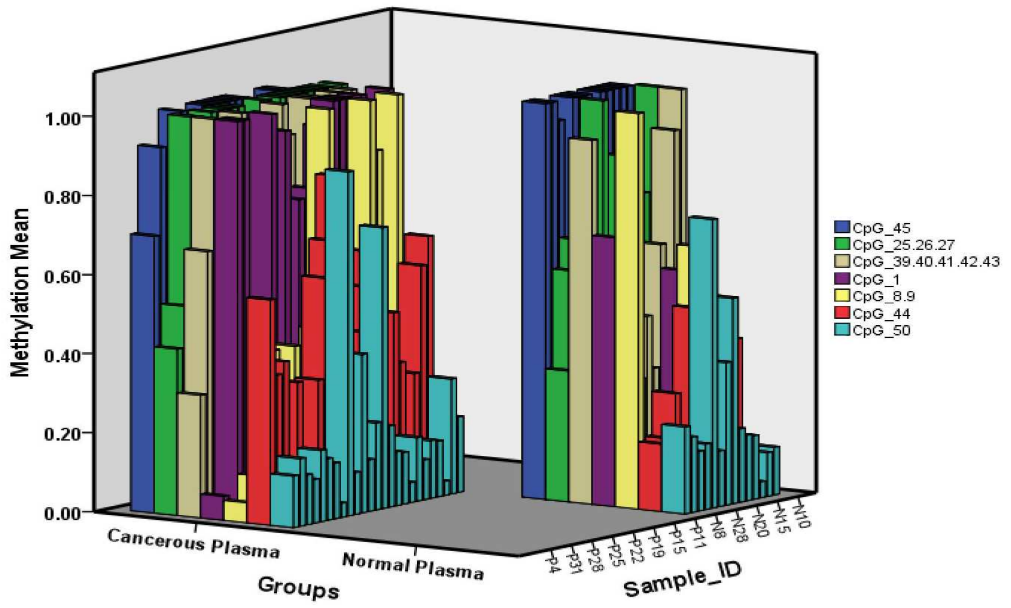

Comparison of informative CpG sites in two groups of plasma samples.

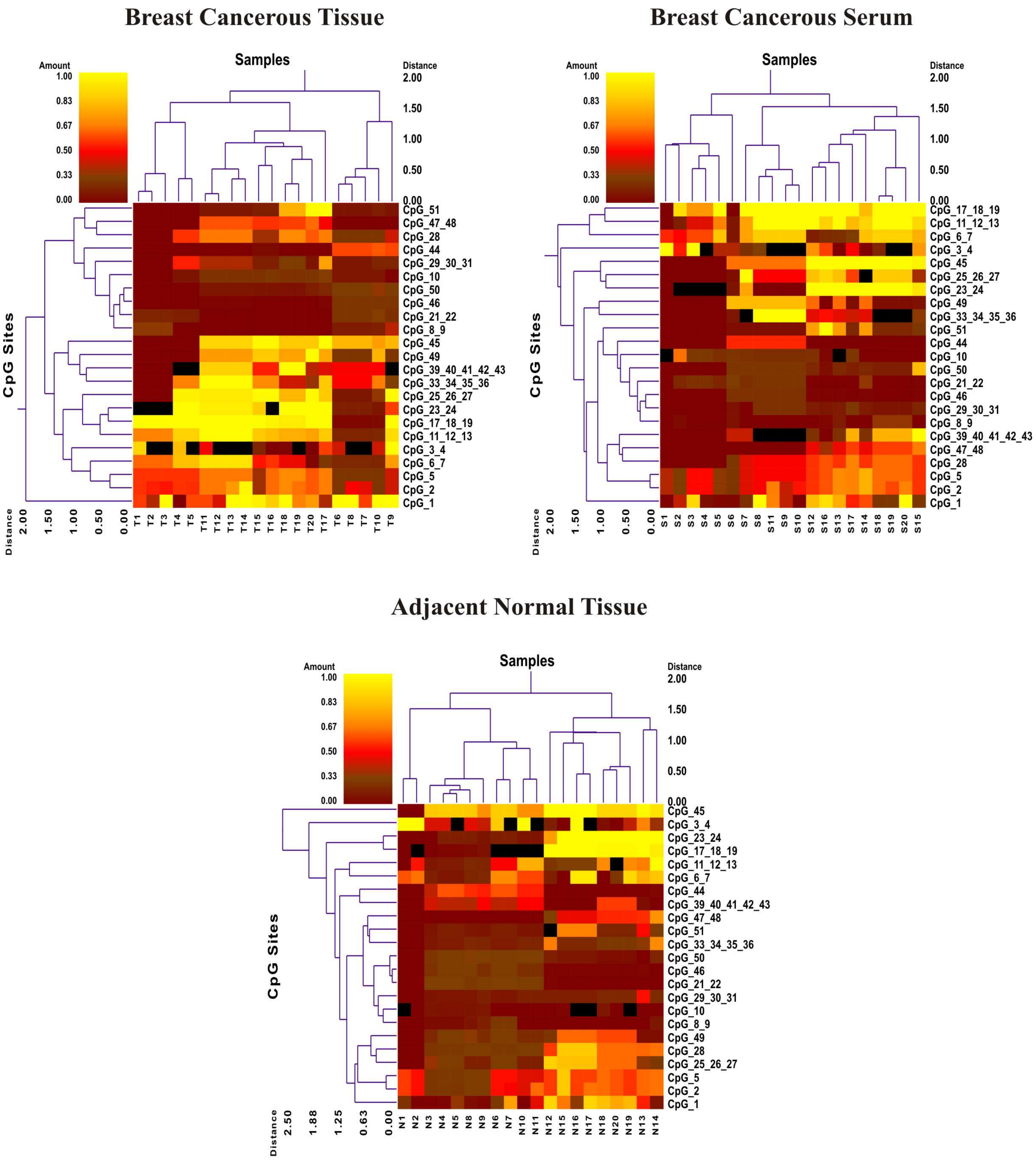

Double dendrogram of *TIMP3* gene: Two-way hierarchical cluster analysis of 60 triple samples (breast cancerous tissue, matched normal tissue serum and samples) from 20 breast cancer patients. (Red clusters indicate 0% methylated, yellow clusters indicate 100% methylated, color gradient between red and yellow indicates methylation ranging from 0-100, and black clusters indicate not analyzed CpG sites).
