# Supplementary material for: Hypermethylation of Tumor Suppressor Genes Involved in Critical Regulatory Pathways for Developing a Blood-Based Test in Breast Cancer
Source: PLoS One. 2011 Jan 24;6(1):e16080. doi: 10.1371/journal.pone.0016080 (PMC3025923; doi:10.1371/journal.pone.0016080)
Supplement: Dataset S2 — Scatterplot Matrix (SPLOM) analysis for mean methylation proportion of 10 genes in triple samples from 20 breast cancer patients (breast cancerous tissue, matched normal tissue and serum samples).Correlation study of the mean methylation proportion of informative CpG sites for ccfDNA in serum versus tumor and normal samples (S: Serum, N: Normal, T: Tumor).Scatterplot Matrix (SPLOM) analysis for mean methylation proportion of 10 genes in 36 plasma samples of breast cancer patients and 30 plasma samples of normal subjects (PDF) [file pone.0016080.s002.pdf]

Dataset S2

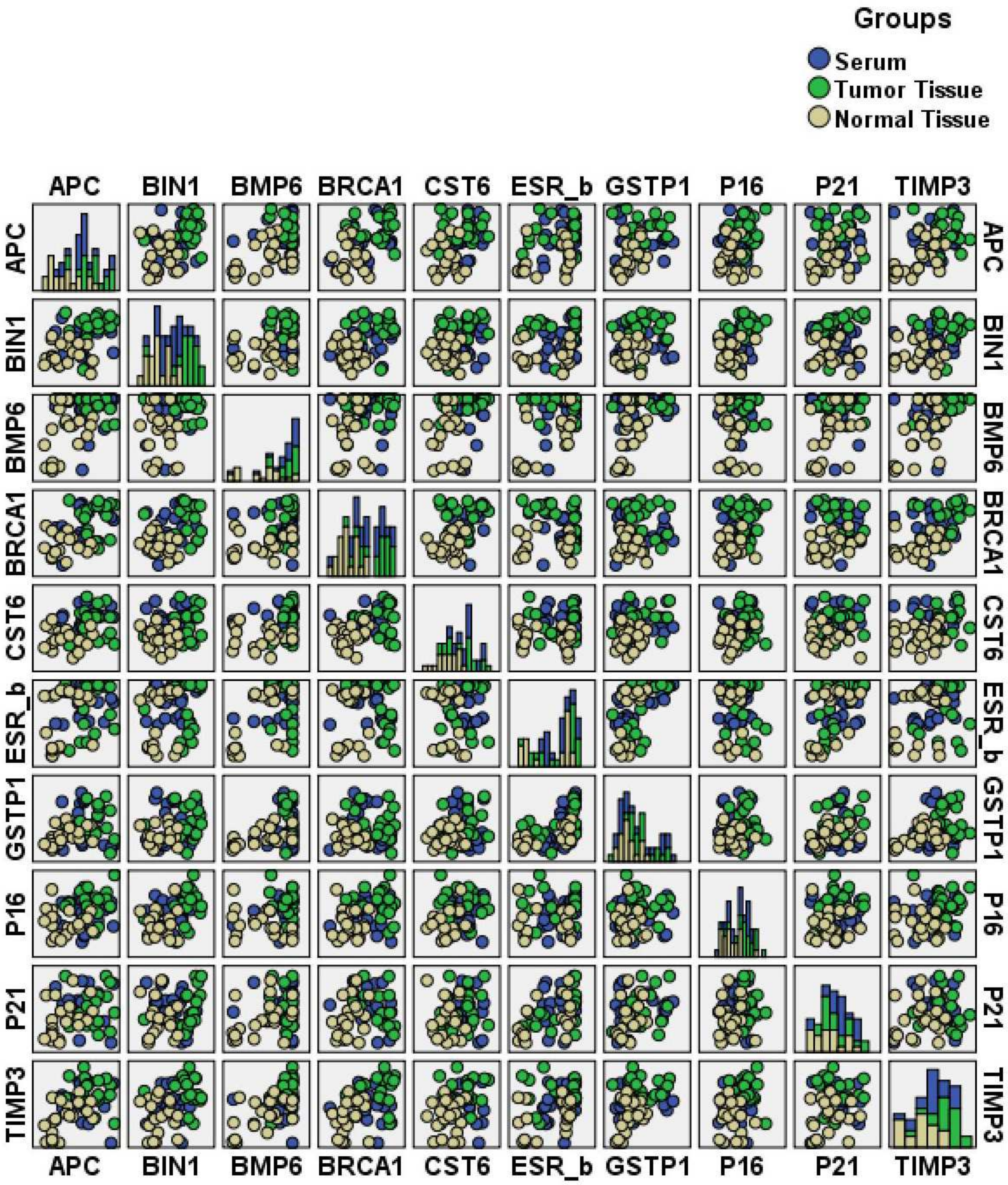

Scatterplot Matrix (SPLOM) analysis for mean methylation proportion of 10 genes in triple samples from 20 breast cancer patients (breast cancerous tissue, matched normal tissue and serum samples).

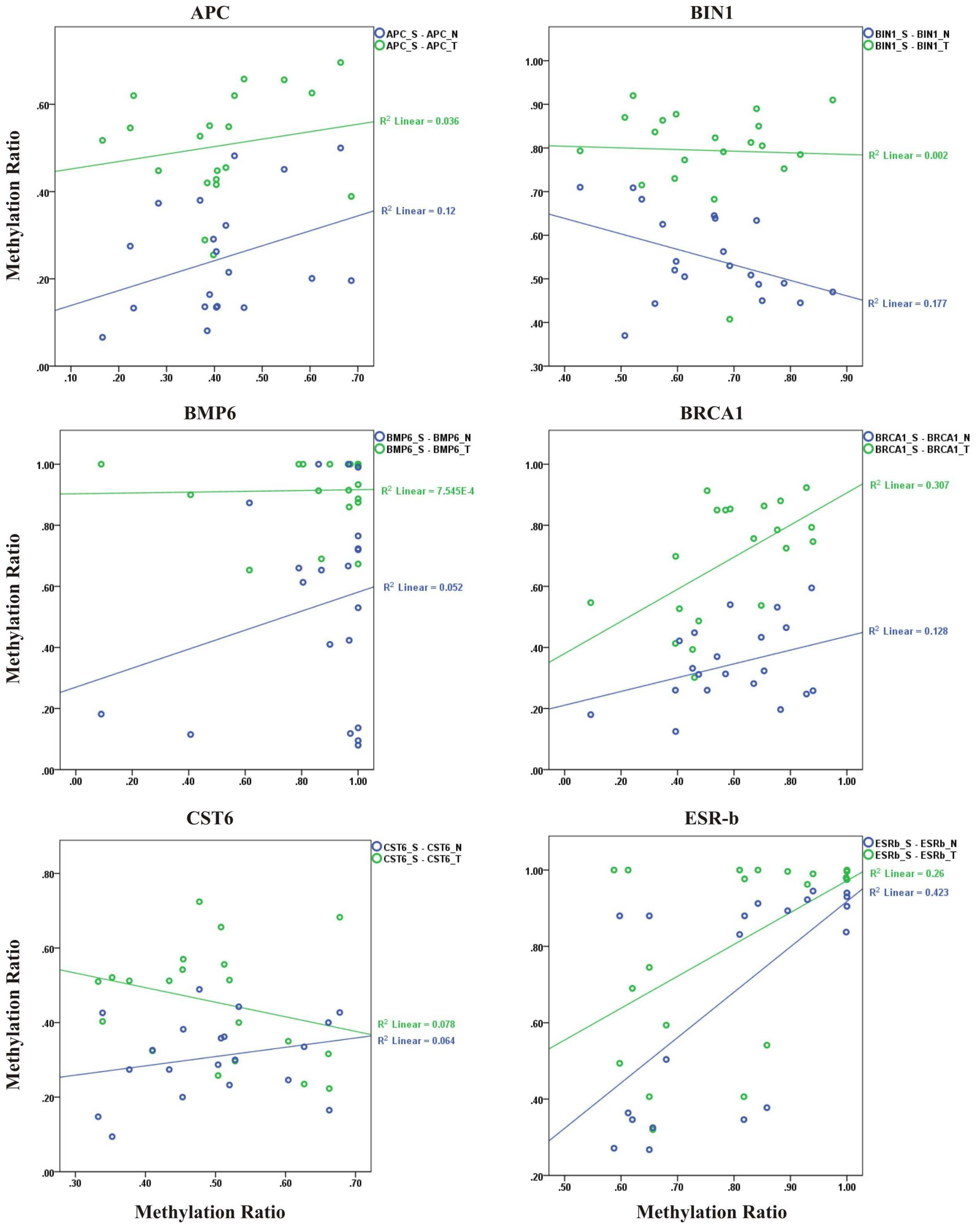

Correlation study of the mean methylation proportion of informative CpG sites for ccfDNA in serum versus tumor and normal samples (S: Serum, N: Normal, T: Tumor).

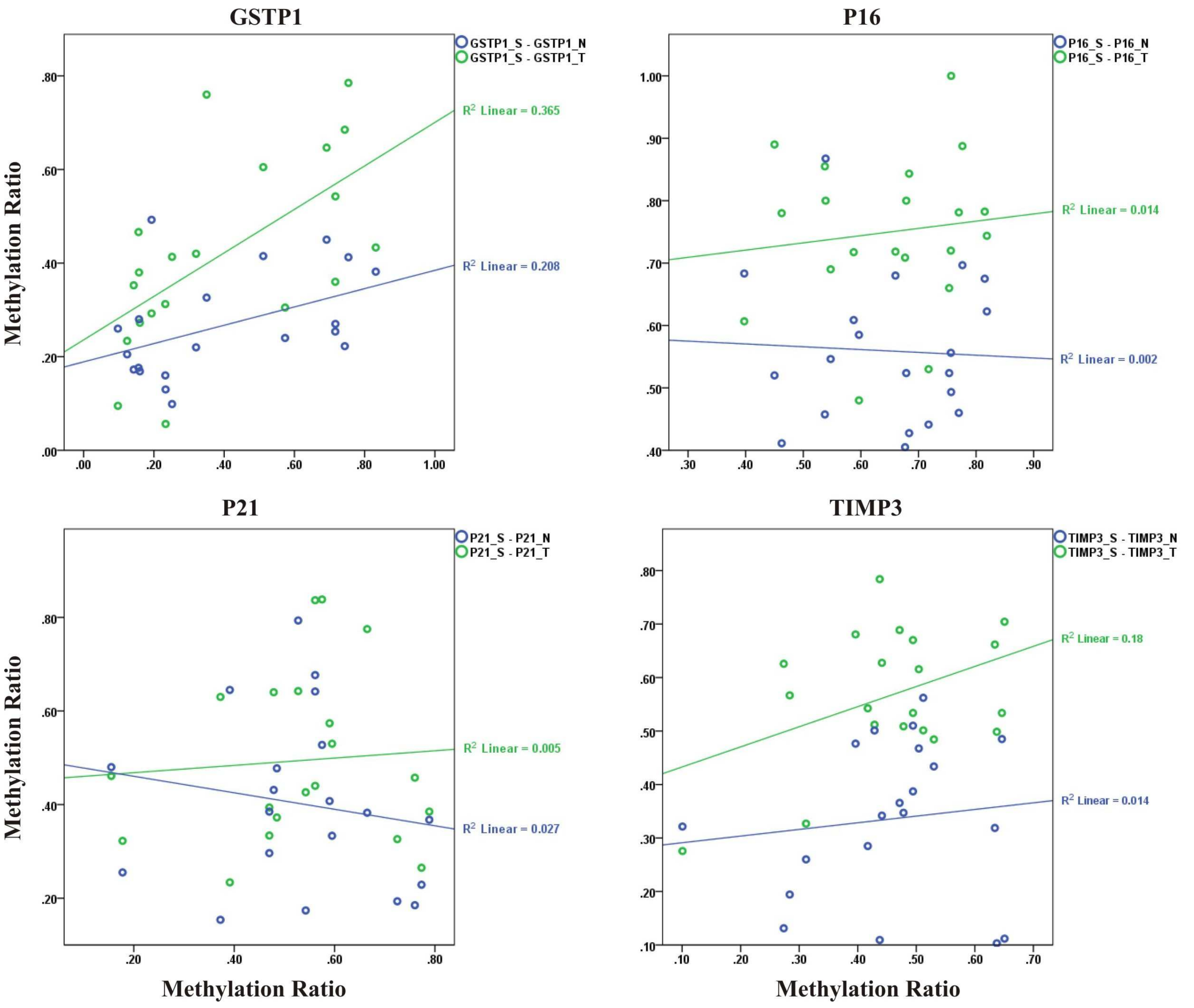

Correlation study of the mean methylation proportion of informative CpG sites for ccfDNA in serum versus tumor and normal samples (S: Serum, N: Normal, T: Tumor).

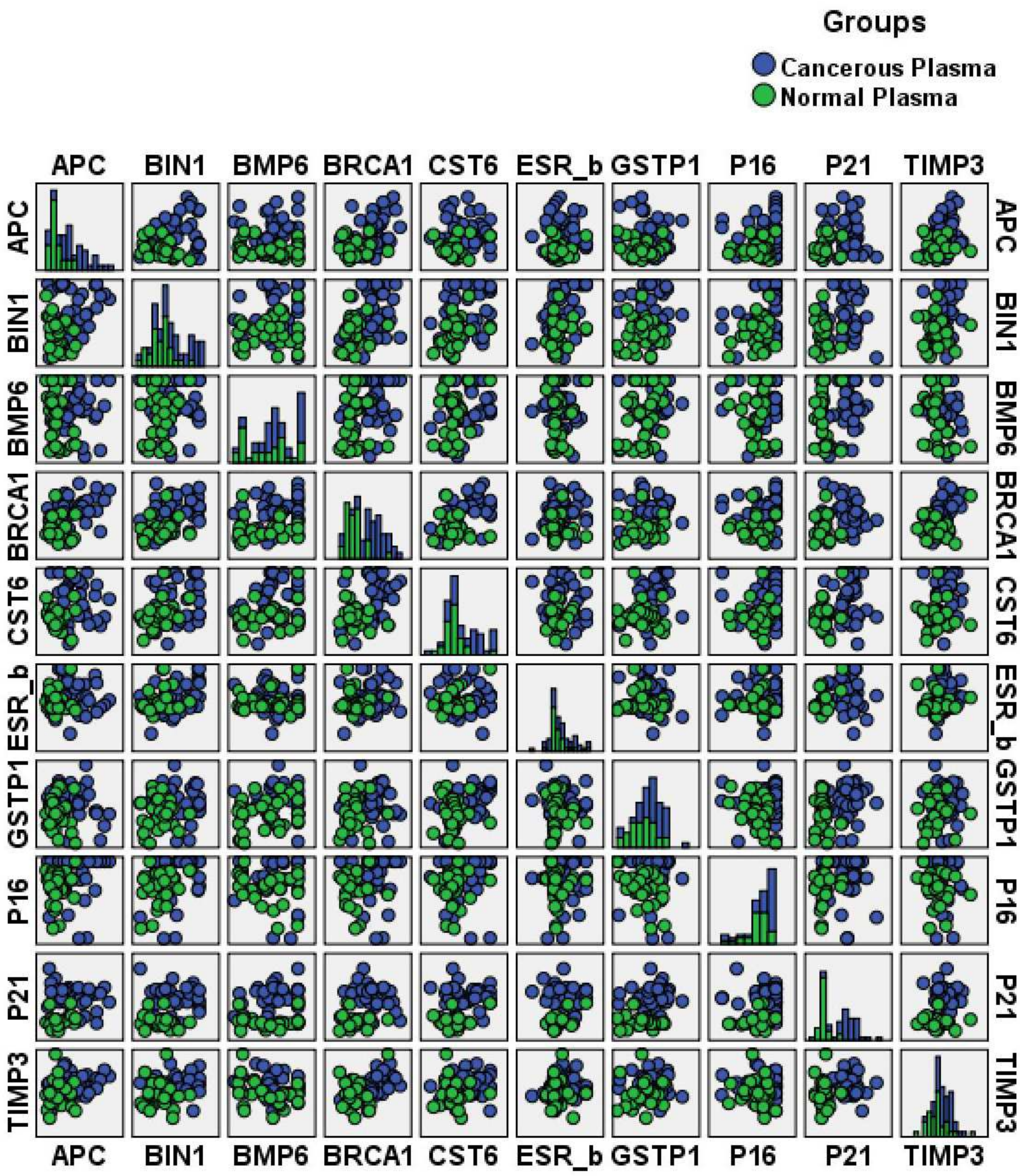

Scatterplot Matrix (SPLOM) analysis for mean methylation proportion of 10 genes in 36 plasma samples of breast cancer patients and 30 plasma samples of normal subjects.
