# Supplementary material for: Hypermethylation of Tumor Suppressor Genes Involved in Critical Regulatory Pathways for Developing a Blood-Based Test in Breast Cancer
Source: PLoS One. 2011 Jan 24;6(1):e16080. doi: 10.1371/journal.pone.0016080 (PMC3025923; doi:10.1371/journal.pone.0016080)
Supplement: Dataset S3 — Correlation study between promoter methylation of 10 studied genes and clinicopathological parameters in 36 plasma and 20 serum samples (PDF) [file pone.0016080.s003.pdf]

Dataset S3

Table 1. Correlation study between promoter methylation of 10 studied genes and clinicopathological parameters in 36 plasma samples.

Variables	Group (No. of cases)	Methylation status of 10 studied genes in *plasma* samples (*P*-value)									
		APC	*BIN1*	*BMP6*	*BRCA1*	*CST6*	*ESR-b*	*GSTP1*	*P16*	*P21*	*TIMP3*
**Age**[a]	<50 (8)<br>≥50 (28)	0.209	0.238	0.577	0.138	0.402	0.834	0.036*	0.098	0.819	0.662
**Histological Grade**[b]	G1 (11)<br>G2 (18)<br>G3 (7)	0.597	0.239	0.788	0.148	0.077	0.891	0.171	0.337	0.399	0.878
**Pathologic Stage**[a]	Early (27)<br>Late (9)	0.927	0.674	0.839	0.265	0.297	0.898	0.273	0.017*	0.351	0.927
**Lymph node involvement**[a]	Positive (19)<br>Negative (17)	0.235	0.295	0.049*	0.401	0.310	0.887	0.466	0.169	0.715	0.173
**ER Marker**[a]	Positive (28)<br>Negative (8)	0.287	0.170	0.115	0.621	0.313	0.361	0.051	0.780	0.819	0.954
**PR Marker**[a]	Positive (23)<br>Negative (13)	0.439	0.339	0.265	0.564	0.222	0.729	0.459	0.163	0.882	1.000

a: Mann-Whitney U Test; **b**: Kruskal-Wallis Test; ***** Significant correlation.
ER: Estrogen receptor; **PR**: Progesterone receptor.

Table 2. Correlation study between promoter methylation of 10 studied genes and clinicopathological parameters in 20 serum samples.

Variables	Group (No. of cases)	Methylation status of 10 studied genes in *serum* samples (*P*-value)									
		APC	*BIN1*	*BMP6*	*BRCA1*	*CST6*	*ESR-b*	*GSTP1*	*P16*	*P21*	*TIMP3*
**Age**[a]	<50 (10)<br>≥50 (10)	0.70	0.705	0.755	0.364	0.545	0.140	0.545	0.151	0.290	0.364
**Histological Grade**[b]	G1 (0)<br>G2 (5)<br>G3 (15)	0.036*	0.150	0.857	0.965	0.239	0.431	0.206	0.407	0.760	0.570
**Pathologic Stage**[a]	Early (12)<br>Late (8)	0.671	0.877	0.061	0.064	0.190	0.089	0.123	0.316	0.354	0.011*
**Lymph node involvement**[a]	Positive (7)<br>Negative (13)	0.874	0.405	0.005*	0.251	0.043*	0.805	0.122	0.322	0.874	0.006*
**ER Marker**[a]	Positive (4)<br>Negative (16)	0.298	0.508	0.807	0.637	0.147	0.256	0.073	0.925	0.887	0.850
**PR Marker**[a]	Positive (10)<br>Negative (10)	0.151	0.597	0.198	0.705	0.705	0.198	0.174	0.174	0.734	0.705

a: Mann-Whitney U Test; **b**: Kruskal-Wallis Test; ***** Significant correlation.
ER: Estrogen receptor; **PR**: Progesterone receptor.
